# Ethics of artificial intelligence in radiology: summary of the joint European and North American multisociety statement

**DOI:** 10.1186/s13244-019-0785-8

**Published:** 2019-10-01

**Authors:** J. Raymond Geis, Adrian Brady, Carol C. Wu, Jack Spencer, Erik Ranschaert, Jacob L. Jaremko, Steve G. Langer, Andrea Borondy Kitts, Judy Birch, William F. Shields, Robert van den Hoven van Genderen, Elmar Kotter, Judy Wawira Gichoya, Tessa S. Cook, Matthew B. Morgan, An Tang, Nabile M. Safdar, Marc Kohli

**Affiliations:** 1 American College of Radiology Data Science Institute, Reston, Virginia USA; 2 Department of Radiology, National Jewish Health, Denver, Colorado USA; 3 Mercy University Hospital, Cork, Ireland; 4 University of Texas MD Anderson Cancer Center, Houston, Texas USA; 5 Department of Linguistics and Philosophy, MIT, Cambridge, Massachusetts USA; 6 Netherlands Cancer Institute, Amsterdam, the Netherlands; 7 Department of Radiology and Diagnostic Imaging, University of Alberta, Edmonton, Alberta Canada; 8 Radiology Department-Mayo Clinic, Rochester, Minnesota USA; 9 Lahey Hospital & Medical Center, Burlington, Massachusetts USA; 10 Pelvic Pain Support Network, Poole, UK; 11 General Counsel, American College of Radiology, Reston, Virginia USA; 12 Center of Law and Internet, Vrije Universiteit Amsterdam, Amsterdam, the Netherlands; 13 Department of Radiology, University Medical Center, Freiburg, Germany; 14 Department of Interventional Radiology, Oregon Health & Science University, Portland, Oregon USA; 15 Department of Radiology and Imaging Sciences, Emory University, Atlanta, Georgia USA; 16 Department of Radiology, University of Pennsylvania, Philadelphia, Pennsylvania USA; 17 Department of Radiology and Imaging Sciences, University of Utah, Salt Lake City, Utah USA; 18 Centre de Recherche du Centre Hospitalier de L’Université de Montréal, Quebec, Canada; 19 Department of Radiology and Biomedical Imaging, UCSF, San Francisco, California USA

**Keywords:** Ethics, Artificial Intelligence, Radiology, Machine Learning, Data

## Abstract

This is a condensed summary of an international multisociety statement on ethics of artificial intelligence (AI) in radiology produced by the ACR, European Society of Radiology, RSNA, Society for Imaging Informatics in Medicine, European Society of Medical Imaging Informatics, Canadian Association of Radiologists, and American Association of Physicists in Medicine.

AI has great potential to increase efficiency and accuracy throughout radiology, but also carries inherent pitfalls and biases. Widespread use of AI-based intelligent and autonomous systems in radiology can increase the risk of systemic errors with high consequence, and highlights complex ethical and societal issues. Currently, there is little experience using AI for patient care in diverse clinical settings. Extensive research is needed to understand how to best deploy AI in clinical practice.

This statement highlights our consensus that ethical use of AI in radiology should promote well-being, minimize harm, and ensure that the benefits and harms are distributed among stakeholders in a just manner. We believe AI should respect human rights and freedoms, including dignity and privacy. It should be designed for maximum transparency and dependability. Ultimate responsibility and accountability for AI remains with its human designers and operators for the foreseeable future.

The radiology community should start now to develop codes of ethics and practice for AI which promote any use that helps patients and the common good and should block use of radiology data and algorithms for financial gain without those two attributes.

**Electronic supplementary material:**

The online version of this article (10.1186/s13244-019-0785-8) contains supplementary material, which is available to authorized users.

## Key points:

- Ethical use of AI in radiology should promote well-being, minimize harm, and ensure that the benefits and harms are distributed among the possible stakeholders in a just manner.
- AI in radiology should be appropriately transparent and highly dependable, curtail bias in decision making, and ensure that responsibility and accountability remains with human designers or operators.
- The radiology community should start now to develop codes of ethics and practice for AI.
- Radiologists will remain ultimately responsible for patient care and will need to acquire new skills to do their best for patients in the new AI ecosystem.

## Introduction

This statement is a condensed version of a statement produced by the ACR, European Society of Radiology, RSNA, Society for Imaging Informatics in Medicine, European Society of Medical Imaging Informatics, Canadian Association of Radiologists, and American Association of Physicists in Medicine. The full version is posted on the web pages of each of these societies. Authors include society representatives, patient advocates, an American professor of philosophy, and attorneys with experience in radiology and privacy in the United States and the European Union.

Artificial intelligence (AI), defined as computers that behave in ways that previously were thought to require human intelligence, has the potential to substantially improve radiology, help patients, and decrease cost [1]. Radiologists are experts at acquiring information from medical images. AI can extend this expertise, extracting even more information to make better or entirely new predictions about patients. Going forward, conclusions about images will be made by human radiologists in conjunction with intelligent and autonomous machines. Although the machines will make mistakes, they are likely to make decisions more efficiently and with more consistency than humans and in some instances will contradict human radiologists and be proven to be correct. AI will affect image interpretation, report generation, result communication, and billing practice [1, 2]. AI has the potential to alter professional relationships, patient engagement, knowledge hierarchy, and the labor market. Additionally, AI may exacerbate the concentration and imbalance of resources, with entities that have significant AI resources having more “radiology decision-making” capabilities. Radiologists and radiology departments will also *be* data, categorized and evaluated by AI models. AI will infer patterns in personal, professional, and institutional behavior. The value, ownership, use of, and access to radiology data have taken on new meanings and significance in the era of AI.

AI is complex and carries potential pitfalls and inherent biases. Widespread use of AI-based intelligent and autonomous machines in radiology can increase systemic risks of harm, raise the possibility of errors with high consequences, and amplify complex ethical and societal issues. Currently there is little experience using AI for patient care in all its demanding and diverse settings. Extensive research remains to be done to understand how to use AI in clinical practice and the operational characteristics they should have. The approach to these issues will be shaped as much by the community’s ethics as by technical factors. Other effects will be more indirect, such as algorithms that make enterprise or public policy decisions or find patterns in the data of large populations to improve public health and our understanding of diseases and treatments.

Radiology’s goal should be to derive as much value as possible from the ethical use of AI, yet resist the lure of extra monetary gain from unethical uses of radiology data and AI. This consensus statement aims to inform a common interpretation of the ethical issues related to using AI in radiology and to inspire radiology AI’s builders and users to enhance radiology’s intelligence in humane ways to promote just and beneficial outcomes while avoiding harm to those who expect the radiology community to do right by them.

People involved with any stage in an AI product’s life cycle must understand it deeply. We have a duty to understand the risks of the products we are using, to alert patients and stakeholders to those pitfalls as appropriate, and to monitor AI products to guard against harm. We have a duty to ensure not just that the use of the product is beneficial overall, but that the distribution of benefits among the possible stakeholders is just and equitable. We should realize that though most changes will be positive, AI will cause inescapable social and economic change, and major social changes such as these are often disproportionately bad for the most vulnerable communities. We must do what we can to ensure that negative consequences are not made worse by unethical distribution.

Radiologists are learning about ethical AI at the same time we invent and implement it. Technological changes in AI, and society’s response to them, are evolving at a speed and scope that are hard to grasp, let alone manage. Our understanding of ethical concerns and our appropriate response to them shift constantly. To do best by our patients and our communities, we have a moral obligation to consider the ethics of how we use and appreciate data, how we build and operate decision-making machines, and how we conduct ourselves as professionals.

## Ethics of Data

The ethics of data are fundamental to AI in radiology and reflect trust in acquiring, managing, and assessing data. Key areas of data ethics include informed consent, privacy and data protection, ownership, objectivity, transparency, the gap between those who have or lack the resources to manage large data sets, and providing meaningful and moral access rights to data [3]. Data “truthfulness” includes understanding how complete and detailed the data are, what information they contain, how accurately they reflect the true physical situation, and measures of variance and bias.

As physicians, radiologists have a moral duty to use the data they collect to serve patients and improve the common good, extract more information about patients and their diseases, and improve the practice of radiology. At the same time, they have a duty to not use data in ways that may harm or adversely influence patients or discriminate against them.

Because developing AI-driven machines today requires well-labeled radiology data, the value of those data and pressures to provide commercial access to them are skyrocketing. In addition to significant good which will come from using these data to improve patient health, there are many ways to unethically capitalize on data, which may harm patients or the common good. Best practices to allow, manage, and contract for that data access are evolving at a rate which outstrips our current knowledge or abilities. This same rapid evolution applies to unethical and questionable practices. One of the greatest challenges is how to thwart those who will attempt to acquire value from unethical data use. Without carefully understanding commercial and technical issues, we are at risk of making substantial and costly mistakes with radiology data.

Bias occurs to some extent with any data set. Common sources of bias potentially promote or harm group-level subsets based on gender, sexual orientation, ethnic, social, environmental, or economic factors. In addition to these traditional sources of bias, radiology AI may be biased by clinically confounding attributes such as comorbidities, and by technical factors such as data set shift and covariate shift due to subtle differences in raw and postprocessed data that come from different scanning techniques. These manifest biases in many ways, each of which deserves research and awareness to minimize the effects on the decisions made by AI models.

When an AI model is implemented, those responsible should be able to answer these questions, and other similar questions, about the ethics of data:

- How will we document and notify patients about how their data are used?
- How should we document data used to train an algorithm, including descriptors for features traditionally associated with bias and discrimination?
- How and by whom are labels generated? What bias might arise from the processes used?
- What kinds of bias may exist in the data used to train and test algorithms?
- What have we done to evaluate how its data are biased, and how it may affect our model?
- What are the possible risks that might arise from biases in the data?
- What steps have we taken to mitigate these biases, and how should users take remaining biases into account?
- Is our method of ground truth labeling appropriate to the clinical use case we are trying to resolve? What are its limitations?

## Ethics of Algorithms and Trained Models

Decision-making is the selection of a belief or a course of action among multiple alternatives, often leading to action. Human decision making is informed by the person’s knowledge, values, preferences, and beliefs. AI chooses alternatives based on features in the input data. For supervised learning, the algorithm chooses that alternative based on prior training to match labels to those data features. Within these labels, human values, preferences, and beliefs may be transferred to the model. This is where human bias may manifest.

Although AI performs well with classification tasks, it is always important to note that an AI product is not human, but rather a computer program envisioned, built, and monitored by humans. Fairness and equality are not AI concepts [4]. Responsibility for these insights falls to humans, who must anticipate how rapidly changing AI models may perform incorrectly or be misused and protect against unethical outcomes, ideally before they occur [5].

To build patient and provider trust in AI, it is important to have as much transparency as possible as to how decisions are made. When errors happen today, we investigate the cause and design systems to eliminate the potential for similar errors in the future. The investigation may address safety, accountability, liability, and technical process changes. Similarly, if an algorithm fails, or contributes to an adverse clinical event, one needs to be able to understand why it produced the result that it did, and how it reached a decision. For a model to be transparent, it should be both visible and comprehensible to outside viewers. How transparent a model should be is debatable. Inappropriate levels of transparency might make it more susceptible to malicious attacks or reveal proprietary intellectual property. Furthermore, imposing a wide definition of transparency could jeopardize privacy by revealing personal data hidden in underlying data sets. The EU General Data Protection Regulation requires data processing to be transparent, although opinions differ about what degree of technical detail is meant by this. Users must be able to explain to the public in plain language how their data will be used to build an intelligent or autonomous tool. Explainability is the ability to explain what happened when the model made a decision, in terms that a person understands. It aims to understand why a model made a particular decision and to appreciate conditions in which the model succeeds and in which it fails. Explainability includes both comprehending technical aspects of algorithm structure and how outputs are presented to the user [6]. Today, models with better explainability usually show worse performance [7]. Current deep learning models have well over 100 million parameters, include techniques which normalize or dropout parameters based on statistical methods, and at least with today’s technology are virtually incomprehensible. Explainable AI is a core area of research [8]. Pinpointing a causative bug in such a system is a daunting task [9]. A more practical approach may be to advocate for visualization and explainability of results, including measures of consistency and generalizability and a mechanism to stop and alert humans when model outputs change or the model’s measurements of certainty fall below a specific level.

Because various AI models are relatively easy to build and train, research and commercial AI-powered solutions may be produced by sometimes naive or unprofessional actors. This increases the importance of extending existing ethical codes in medicine, statistics, and computer science to consider situations specific to radiology AI [3, 10, 11].

Adversarial attacks are well-known in other AI domains, and the radiology AI community is becoming aware of them [12–15]. Although potential solutions may exist, radiology as a field has no defense against such attacks. This weakness must be acknowledged and addressed. When an AI model is implemented, those responsible for any part of its life cycle should be able to answer these and other similar questions about the Ethics of algorithms:

- Are we able to explain how our AI makes decisions or at least reliably predict the results of our AI analysis in known data sets?
- How do we protect against malicious attacks on AI tools and data?
- How do we create sustainable version control for AI data, algorithms, models, and vended products?
- How will we minimize the risk of patient harm from malicious attacks and privacy breaches?
- How will we evaluate trained models before clinical application, for clinical effectiveness, ethical behavior, and security?
- How will we monitor AI models in clinical workflow to ensure they perform as predicted and that performance does not degrade over time?

## Ethics of Practice

Radiology AI is a complex ecosystem of clinical care, technological and mathematical advances, and business and economics. Moral behavior, doing the right thing, can be intellectually uncertain. Popular media provide daily accounts of how technical innovation crosses into unprincipled activities, which, even if unintentional, may cause considerable harm to patients, society, and individual and business reputations. Conscientious ethical values should guide decisions about when to apply AI, define metrics to describe appropriate and responsible AI, and recognize and alert the community to unethical AI.

Automation bias is the tendency for humans to favor machine-generated decisions, ignoring contrary data or conflicting human decisions. Automation bias leads to errors of omission and commission. Omission errors occur when humans fail to notice, or disregard, the failure of an AI tool. High decision flow rates, in which decisions are swiftly made on radiology examinations being read rapidly by a radiologist, predispose to omission errors. This is compounded by AI decisions based on features that are too subtle for humans to detect. Commission errors occur when one erroneously accepts or implements a machine’s decision despite other evidence to the contrary. Automation bias risks may be magnified in resource-poor populations because there is no local radiologist to veto the results.

To what degree can physicians delegate the task of diagnosing medical conditions to intelligent or autonomous systems without exposing themselves to increased liability for malpractice if the system makes an error? Such questions regarding AI-caused harm will arise with ever-increasing frequency as these tools become pervasive. Sources of liability may occur from issues such as data privacy, contracts, negligence, criminal behavior, vicarious liability, or insurance [16, 17]. AI developers ultimately need to be held to the same “do no harm” standard as physicians. Although liability ultimately falls to humans, determining legal responsibility when an AI system’s decision results in harm is still in flux. It may be difficult to determine to what extent data, the AI algorithm itself, and how it is used are responsible for harm. Different liability models may arise for different settings and forms of AI. Over time, a risk liability system will evolve.

Smaller or resource-poor hospitals and academic departments may lack the technology, skills, and resources to manage complex AI systems. Almost certainly some radiology AI will be proprietary, developed by large academic or private healthcare entities, insurance companies, or large companies with data science expertise but little historical radiology domain knowledge. This may exacerbate disparities in access to radiology AI.

As radiology incorporates autonomous and intelligent AI products into widespread, demanding clinical practice, those responsible should be able to answer these and other similar questions about the ethics of this new practice paradigm:

- What are the patient and provider risks associated with this AI implementation, and what level of human oversight is necessary to mitigate these risks?
- What education and skills are needed to decide whether to apply AI to our patients and to safely and effectively use it when appropriate?
- How do we ensure that testing data accurately reflects the targeted clinical cohort?
- What processes should we implement to monitor the impact (outcomes, privacy, and unintended discrimination) of AI on our patients and providers (automation bias)?
- How do we continuously and actively monitor AI-driven autonomous and intelligent tools to verify they are working as expected in clinical care?
- What guardrails should we use to determine when, and more importantly when not, to implement autonomous or intelligent mechanical agents?

## Conclusion

Ethical use of AI in radiology should promote well-being, minimize harm, and ensure that the benefits and harms are distributed among the possible stakeholders in a just manner. It should respect human rights and freedoms, including dignity and privacy. It should be appropriately transparent and highly dependable, curtailing bias in decision making while ensuring that responsibility and accountability remain with human designers or operators.

The radiology community should start now to develop codes of ethics and practice for AI. These codes should promote any use which helps patients and the common good and block use of radiology data and algorithms for financial gain without those two attributes. Establishing these regulations, standards, and codes of conduct to produce ethical AI means balancing the issues with appropriate moral concern. Ensuring ethical AI requires a desire to gain trust from all parties involved. To ensure the safety of patients and their data, AI tools in radiology need to be properly vetted by legitimately chosen regulatory boards before they are put into use. This requires both radiology-centric AI expertise and technology to verify and validate AI products.

Regulations, standards, and codes of conduct must be agreed upon and continually updated. Key to these codes of conduct will be a continual emphasis for transparency, protection of patients, and vigorous control of data versions and uses. Continuous postimplementation monitoring for unintended consequences and loss of quality must be enforced, with protocols in place for determining causes and implementing corrective action.

New ethical issues will appear rapidly and regularly, and our appreciation of them will change over time. Thus, although it is important to consider the ethics of AI in radiology now, it also will be important to reassess the topic repeatedly as our understanding of its impact and potential grows and to return to the AI tools being used in radiology to assess whether they meet the updated regulations and standards.

Radiologists will remain ultimately responsible for patient care and will need to acquire new skills to do their best for patients in the new AI ecosystem. The radiology community needs an ethical framework to help steer technological development, influence how different stakeholders respond to and use AI, and implement these tools to make best decisions and actions for, and increasingly with, patients. We hope that this statement clarifies the core principles upon which this framework ought to be based in each community.

## Additional file

Additional file 1: Ethics of AI in Radiology European and North American Multisociety Statement. (PDF 459 kb)

## Data Availability

All data generated or analysed during this study are included in this published article.

## References

[1] Kohli M, Prevedello LM, Filice RW, Geis JR (2017) Implementing machine learning in radiology practice and research. AJR Am J Roentgenol:1–7 10.2214/AJR.16.17224 10.2214/AJR.16.17224 28125274

[2] Erickson BJ, Korfiatis P, Akkus Z, Kline TL (2017) Machine learning for medical imaging. Radiographics 37:505–515 10.1148/rg.2017160130 10.1148/rg.2017160130 PMC5375621 28212054

[3] Mittelstadt BD, Floridi L (2016) The ethics of big data: current and forseeable issues in biomedical contexts. Sci Eng Ethics 22:303–341 10.1007/s11948-015-9652-2 10.1007/s11948-015-9652-2 26002496

[4] IEEE Standards Association. IEEE Global Initiative Ethically Aligned Design, Version 2 (EADv2). Institute of Electrical and Electronics Engineers. Available via: https://standards.ieee.org/content/dam/ieeestandards/standards/web/documents/other/ead_v2.pdf. Accessed 23 Aug 2019

[5] O’Neil C (2016). Weapons of Math Destruction: How Big Data Increases Inequality and Threatens Democracy.

[6] Gilpin LH, Bau D, Yuan BZ, Bajwa A, Specter M, Kagal L (2018) Explaining explanations: an overview of interpretability of machine learning. ArXiv180600069 Cs Stat

[7] Schönberger D (2019). Artificial intelligence in healthcare: a critical analysis of the legal and ethical implications. Int J Law Inf Technol.

[8] Defense Advanced Research Projects Agency. Explainable artificial intelligence. Available via: https://www.darpa.mil/program/explainable-artificial-intelligence. Accessed 17 Feb 2019.

[9] Responsible AI Practices. In: Google AI. Available via: https://ai.google/education/responsible-ai-practices/. Accessed 17 May 2019

[10] Floridi L, Taddeo M (2016) What is data ethics. Philos Trans A Math Phys Eng Sci 28;374:20160360. 10.1098/rsta.2016.0360 PMC5124072 28336805

[11] Li Y, James L, McKibben J (2016) Trust between physicians and patients in the e-health era. Technol Soc 46:28–34. 10.1016/j.techsoc.2016.02.004

[12] Mirsky Y, Mahler T, Shelef I, Elovici Y (2019) CT-GAN: malicious tampering of 3D medical imagery using deep learning. 2019. Available via: https://arxiv.org/abs/1901.03597. Accessed 23 Aug 2019

[13] Chuquicusma MJM, Hussein S, Burt J, Bagci U (2017) How to fool radiologists with generative adverssarial networks? A visual Turing test for lung cancer diagnosis. ArXiv171009762 Cs Q-Bio

[14] Finlayson SG, Chung HW, Kohane IS, Beam AL (2018) Adversarial attacks against medical deep learning systems. ArXiv180405296 Cs Stat

[15] Kim H, Jung DC, Choi BW (2019) Exploiting the vulnerability of deep learning-based artificial intelligence models in medical imaging: adversarial attacks. J Korean Soc Radiol 80:259–273 10.3348/jksr.2019.80.2.259

[16] Barwell BSL-E (2018) Legal liability options for artificial intelligence | Lexology. Available via: https://www.lexology.com/library/detail.aspx?g=6c014d78-7f4c-4595-a977-ddecaa3a12e4. Accessed 8 July 2019

[17] Kingston J (2018) Artificial intelligence and legal liability. ArXiv180207782 Cs

