# Supplementary material for: Ethics of artificial intelligence in radiology: summary of the joint European and North American multisociety statement
Source: Insights Imaging. 2019 Oct 1;10:101. doi: 10.1186/s13244-019-0785-8 (PMC6768929; doi:10.1186/s13244-019-0785-8)
Supplement: Supplementary file 1 — Ethics of AI in Radiology European and North American Multisociety Statement. (PDF 459 kb) [file 13244_2019_785_MOESM1_ESM.pdf]

2019

Ethics of AI in Radiology

European and North American Multisociety Statement

Ethics of AI in Radiology: European and North American Multisociety Statement

Summary 6

Introduction 8

About this Statement 10

Ethics of Data 11

Clinical radiology data 12

Business operational and analytic data 13

Pre-training, synthetic, and augmented data 13

Raw image data 14

Data ownership 14

Data sharing and data use 16

Data privacy 20

Bias and data 21

Data labeling and ground truth 23

Ethics of Algorithms and Trained Models 25

Algorithm selection 26

Algorithm training 27

Model evaluation and testing 27

Transparency, interpretability, and explainability 29

Open source software 31

Replicability 31

Algorithm bias 31

Security 32

Ethics of Practice 32

Computer - human interaction: Keeping humans in the loop 33

Education 35

Automation bias 35

Patient preferences 36

Traceability 37

AI and workforce disruption 37

Resource inequality 38

Liability 39

Conflicts of interest 40

Conclusion 41
Definitions 43
References 44

Authors

J. Raymond Geis, MD
Adjunct Associate Professor of Radiology, National Jewish Health, Denver, CO
Senior Scientist, American College of Radiology Data Science Institute, Reston, VA
Clinical Assistant Professor of Radiology, University of Colorado School of Medicine, Aurora, CO
Representing ACR

Adrian Brady, FFRRCSI, FRCR, FRCPC
Consultant Radiologist, Mercy University Hospital, Cork, Ireland
Chair of ESR Quality, Safety & Standards Committee.
Representing ESR

Carol C. Wu, MD
Associate Professor
University of Texas MD Anderson Cancer Center, Houston, TX
Representing RSNA

Jack Spencer, PhD
Associate Professor
MIT, Department of Linguistics and Philosophy

Marc Kohli, MD
Medical Director of Imaging Informatics, UCSF Health
Associate Professor of Radiology and Biomedical Imaging, UCSF, San Francisco, CA
Representing SIIM

Erik Ranschaert, MD PhD
Radiologist, ETZ Hospital, Tilburg, the Netherlands.
President, European Society of Medical Imaging Informatics
Representing EuSoMII

Jacob L. Jaremko, MD, FRCPC, PhD
Associate Professor, Alberta Health Services
Chair of Diagnostic Imaging, Department of Radiology and Diagnostic Imaging, University of Alberta, Edmonton, Alberta, Canada
Representing CAR

Steve G. Langer, PhD
Chair, Enterprise Radiology Architecture and Standards, Co-Chair Radiology Informatics Laboratory
Professor of Diagnostic Imaging and Informatics, Radiology Department-Mayo Clinic, Rochester, MN
Representing AAPM

Andrea Borondy Kitts, MS MPH
Patient Outreach & Research Specialist
Lahey Hospital & Medical Center, Burlington, MA
Patient Advocate

Judy Birch, B.Ed
Pelvic Pain Support Network, Poole, UK
Patient Advocate
Representing ESR PAG

William F. Shields, JD, LLM
General Counsel
American College of Radiology

Robert van den Hoven van Genderen, Phd, MSC, LLM,
Director, Center of Law and Internet, Vrije Universiteit Amsterdam
Managing Partner, Switchlegal international Lawyers
Chairman, Netherlands Association for AI and Robotlaw

Elmar Kotter, MSc MD MBA
Deputy Director and Head of IT, Department of Radiology, University Medical Center, Freiburg, Germany
Associated Professor of Radiology, University of Freiburg, Freiburg, Germany
Chair, ESR eHealth and Informatics Subcommittee
Vice President, European Society of Medical Imaging Informatics (EuSoMII)
Representing ESR

Judy Wawira Gichoya MBChB MS
Department of Interventional Radiology, Oregon Health & Science University, Portland, OR
Representing ACR

Tessa S. Cook, MD PhD
Assistant Professor of Radiology
Fellowship Director, Imaging Informatics
Co-Director, Center for Practice Transformation
University of Pennsylvania, Philadelphia PA
Representing ACR

Matthew B. Morgan, MD MS
Associate Professor
Department of Radiology and Imaging Sciences
University of Utah
Representing RSNA

An Tang, MD MSc
Radiologist, Centre Hospitalier de l'Université de Montréal
Researcher, Centre de Recherche du Centre Hospitalier de L'Université de Montréal, Quebec, Canada
Representing CAR

Nabile M. Safdar, MD MPH
Associate Chief Medical Information Officer, Emory Healthcare
Vice Chair of Informatics, Dept. of Radiology and Imaging Sciences, Emory University, Atlanta, GA
Representing RSNA

Summary

Artificial intelligence (AI), defined as computers that behave in ways that, until recently, were thought to require human intelligence, has the potential to substantially improve all facets of radiology [1]. AI is complex, has numerous potential pitfalls, and is inevitably biased to some degree. This statement aims to inform a common interpretation of the issues and the ultimate goals of using AI-based intelligent and autonomous machines in radiology. Technology tilts people in certain directions. We hope to tilt the radiology approach to this powerful technology in the correct direction up front, and describe a path to aspire radiology AI's builders and users to enhance radiology's intelligence in humane ways to promote just and beneficial outcomes, while avoiding harm to those who expect us to do right by them.

Intelligent and autonomous machines will make substantial clinical and workflow decisions in radiology. While this will mature into reliable and robust infrastructures, currently no one has substantial experience using such machines for rigorous patient care in diverse settings. This gives rise to potential errors with high consequences. We hypothesize what is important when using such machines, such as transparency and explainability, and we have rudimentary experience managing AI tools. We have much to learn, and extensive research remains to be done to understand how to use these machines in widespread clinical practice, and the operational characteristics they should have.

Because developing AI-driven machines today requires massive amounts of well-labeled radiology data, the value of those data is skyrocketing and the drive to provide commercial access to radiology data will become overwhelming. Currently the best ways to allow, manage, and contract for that data access are evolving at a rate which outstrips our current knowledge or abilities. We are at risk of making expensive and calamitous mistakes with radiology data.

In addition to the significant good which will come from using these data to make better predictions and improve patient health, there are many ways to unethically capitalize on data which may harm patients, other cohorts, or the common good. Limiting radiology AI to ethical uses means leaving money on the table. One of our greatest challenges is how to thwart those who will attempt to acquire this value.

Patients, radiologists, and other cohorts in the radiology community are at risk of being engulfed by digital surveillance, and categorized and manipulated by intelligent and autonomous machines. Radiology and other medical data could be weaponized in the same way as data from non-medical sources.

Radiologists are experts at acquiring information from radiology images. AI can extend this expertise, extracting even more information to make better or entirely new predictions about patients. At the same time, we see daily the ways that AI potentially hurts both user and those on whom it is used, and harms the reputations of organizations and professions. People involved with each stage in an AI product's life cycle must understand it deeply. They have a duty to understand the risks of the products they are using, to alert patients and stakeholders to those pitfalls as appropriate, and to monitor AI products to guard against harm. They have a duty to ensure not just that the use of the product is beneficial overall, but that the distribution of benefits among the possible stakeholders is just and equitable. We should realize that though most changes will be positive, AI will cause inescapable social and economic change, and major social changes such as these are often disproportionately bad for the most vulnerable communities. We must do what we can to avoid negative consequences and ensure that unavoidable or unexpected negative consequences are not made worse by unethical distribution.

AI has dramatically altered the value, use, and potential of misuse of radiology data. Radiologists have a moral duty to use the data they collect to improve the common good, extract more information about patients and their diseases, and improve the practice of radiology. At the same time, they have a duty to not use data in ways that may harm or adversely influence patients or discriminate against them.

Bias occurs to some extent with any dataset. This manifests in many ways, each of which deserves research and awareness to minimize the effects on the decisions made by AI models.

The radiology community and relevant stakeholders should start now to develop codes of ethical practice for AI. Ensuring ethical AI requires a desire to gain trust from all involved. Effective regulations, standards, and codes of conduct will need to balance technical, clinical, population health, and commercial motivations with appropriate moral concern. Agencies will need to have the authority to enforce them. Key to these codes of conduct will be a continual emphasis on transparency, protection of patients, and vigorous control of data and algorithm versions and uses. AI tools will need to be monitored continuously and carefully to ensure they work as expected, and that the decisions they make enable optimal and ethical patient care.

The radiology community is learning about ethical AI while simultaneously trying to invent and implement the technology. This is occurring amid technological evolution at a speed and scope which are difficult to comprehend. AI will conceivably change radiologists' roles and positions, revolutionize how decisions are made about radiology exams, and transform how radiologists relate to patients and other stakeholders.

Introduction

This statement arises from the multi-national radiology community's desire to examine the ethics and code of behavior for AI in radiology. Our goals are to foster trust among all parties in radiology AI doing the right thing for patients and the community, and to see ethical aspirations applied to all aspects of AI in radiology. To encourage research on these topics, we describe ethical issues associated with designing and using autonomous and intelligent systems in radiology for the greater good of patients, understanding how they work, and avoiding harm by their use. To a lesser extent, we examine objectives for regulations and codes of conduct for this field. We illustrate the medical, cultural, and commercial factors which affect the confluence of AI, radiology, and ethics.

Radiologists have years of specialized training to acquire the knowledge and skills necessary to analyze radiology images to discover intimate and often life-altering information about what is occurring inside their patients' bodies. Patients, other customers, and the public rely on radiologists to make decisions based on imaging examinations. This unique decision-making capability creates a hierarchy of authority between radiologists and those who rely on them. Radiologists' professional code of ethics aims to ensure that the authority wielded by radiologists leads to moral outcomes. AI and machine learning (ML) are statistical methods that will increase the information radiologists can extract from radiology examinations, enrich radiology decision-making, and improve patient care in radiology.

Going forward, conclusions about images will be made not just by human radiologists, but in conjunction with intelligent machines. In some instances, the machines may make better decisions, make them more quickly or efficiently, or contradict the human radiologists. AI will affect image interpretation, the what and how of reporting, how we communicate, and how we bill for services[1, 2]. AI has the potential to alter professional relationships, patient engagement, knowledge hierarchy, and the labor market. Additionally, AI may exacerbate the concentration and imbalance of resources, with entities that have significant AI resources having more "radiology decision-making" capabilities. Radiologists and radiology departments will also *be* data, categorized or evaluated by AI models. AI will deduce patterns in personal, professional, and institutional behavior. AI is transforming traditional thinking about radiology data how 'truthful' and 'ethical' are the data, who owns them, who has access to them, who knows what, and how they use that power.

While AI promises to improve quality, patient outcomes, and efficiency, and decrease costs, it will also produce new possibilities, consequences, and questions for both patients and the radiology community. These issues will be shaped as much by the community's ethics as by technical factors. Other effects will be more indirect, such as algorithms that make enterprise

or public policy decisions, or find patterns in the data of large populations to improve public health and our understanding of diseases and treatments.

Given its potential benefits, we feel there is a duty to actively pursue AI and use it to improve radiology. But to ensure the safety of patients and their data, AI tools in radiology need to be properly vetted by legitimately chosen regulatory boards before they are put into use. New ethical issues will appear rapidly and regularly, and our appreciation of them will change over time. Thus, while it is important to consider the ethics of AI in radiology now, it also will be important to reassess the topic repeatedly as our understanding of its impact and potential grows and to return to the AI tools being used in radiology to assess whether they meet the updated regulations and standards.

At the start, most radiology AI will consist of intelligent clinical decision support models integrated into radiologists' workflow, such as measurement tools or computer assisted detection (CAD) already in use today. Increasingly, however, AI agents will be autonomous, and make decisions and initiate actions on their own, without radiologists' supervision.

Extrapolating from other industries and looking far into the future, AI-enabled radiology will mature into a complex environment containing dynamic networked systems[3]. These intricate webs of autonomous algorithms will be like multiple radiologists each making decisions about one focused portion of an exam. Depending on their consensus, they will then pass the examination to other groups of autonomous algorithms, which, in turn will make decisions on other parts of the exam. Complex, web-like cascades of these decision-making computers will accept and transmit information to each other, and the decisions made will change over time.

Dynamic networked systems for radiology have barely been conceived, and are years from being designed or built. Much remains to be learned about how to assemble such systems in a robust, secure, accurate, and reliable fashion, or how to understand their "behavior", or processing logic.

Radiologists will remain ultimately responsible for what happens to patients and will need to acquire new skills to manage these ecosystems and ensure patients' well-being. The radiology community needs an ethical framework to help steer technological development, influence how different stakeholders respond to and use AI, and implement these tools to make best decisions and actions for, and increasingly with, patients. We recommend that a committee representing each of the relevant stakeholders be assembled in the very near future and tasked with producing that framework.

Because some AI models are relatively easy to build and train, research and commercial AI-powered solutions are being produced by a large number of sometimes naive or unprofessional actors. This increases the importance of extending existing ethical codes in medicine, statistics, and computer science to consider situations specific to radiology AI[4–6].

Many fields outside medicine, and medical societies, are evaluating the ethics of AI. Recent *New England Journal of Medicine* and *Journal of the American Medical Association* articles describe both the promise of AI[7] and the acute need to address the potential for bias and questions about the fiduciary relationship between patients and AI[8, 9]. Leaders in computer science and engineering, including the Institute of Electrical and Electronics Engineers (IEEE), the Association for Computing Machinery (ACM), Future of Life Institute, and governmental bodies such as the European Commission's Group on Ethics in Science and New Technologies, are updating their recommendations and guidance[10–13]. Many other professional, regulatory and academic bodies have published, or are in the process of preparing statements about ethical use of AI. Depending on the focus of the publishing body, the details of these statements concentrate on varying aspects of AI deployment and usage, but the commonality of principles among these statements is:

1. Promote well-being, minimize harm, and ensure that the benefits and harms are distributed among the possible stakeholders in a just manner.
2. Respect human rights and freedoms, including dignity & privacy.
3. Be transparent and dependable, curtailing bias and decision, while ensuring that the locus of responsibility and accountability remains with their human designers or operators.

About this Statement

This statement is a joint effort by the American College of Radiology, European Society of Radiology, Radiology Society of North America, Society for Imaging Informatics in Medicine, European Society of Medical Imaging Informatics, Canadian Association of Radiologists, and American Association of Physicists in Medicine. The core writing team includes an American philosopher, North American and European radiologists, imaging informaticists, medical physicists, patient advocates, and attorneys with experience in radiology in the U.S. and EU.

In developing this statement, we reviewed current ethics literature from computer science and medicine, as well as historical ethical scholarship, and material related to the ethics of future scenarios. In the interest of efficiency, our statement focuses on North America and Europe. We realize that other regions may have values and ethics which both overlap and differ.

This statement is intended to be aspirational rather than prescriptive. We aim to provide an approach to the ethics of AI that is easy to understand and implement. We expect this topic will change rapidly as technology and data science advances, and new legal approaches and liability descriptions evolve to deal with automated decision making. California's new data privacy law[14, 15] and the European Union's GDPR[16] and proposed Civil Law Rules on Robotics[17] are harbingers of such legislation. People who build commercial and generalizable radiology AI tools need instructive ethical guidance; this statement will help inform future groups charged with composing such regulations. This statement provides a baseline for recommendations and ethical questions to consider when planning to implement AI in radiology.

Ethical use of AI in radiology must respect the ethical principles of humanity, the protection of human subjects of biomedical and behavioral research[18], and mandates of public reason. Some of radiology's ethical issues are deep and difficult; in those cases, we try to raise awareness of what we regard to be the most pressing ethical issues, explain how the issues specifically involve radiology, and suggest factors the radiology community should consider. Where we identify ethical issues that pertain specifically to radiology with answers that are sufficiently clear, we will suggest strategies.

This statement is structured using a process described by Floridi et al.,[4]. Ethics topics are divided into ethics of data, ethics of algorithms, and ethics of practice.

Ethics of Data

The ethics of data are fundamental to AI in radiology. Key areas of data ethics include informed consent, privacy and data protection, bias and data "truthfulness," ownership, objectivity, transparency, and the gap between those who have or lack the resources to use large datasets. Other data issues include bias against group-level subsets based on gender, ethnic, or economic groups, the importance of trust in assessing data ethics, and providing meaningful and moral access rights to data[5].

AI has dramatically altered our perception of radiology examinations and associated data including their value, how we use them and how they may be misused. In addition to understanding AI, radiologists have a duty to understand the data. Radiologists and the radiology community have a moral duty to use the data they collect to improve the common good, extract information about patients and their diseases, and improve the practice of radiology. Radiologists are ethically obligated to make their data useful to the patients it was collected from.

Clinical radiology data

An imaging examination typically consists of image data and associated labels[19].

Image data are produced by a piece of imaging equipment, and subsequently processed to generate human-viewable and -interpretable images. The raw data produced by the imaging modality cannot be interpreted by humans, and must be converted into collections of pixels, which we commonly refer to as an image. Pixels are the “dots” that form the images that humans evaluate. While the pixel data are saved, and often combined with additional meta-data, raw data is usually purged after several days. In some instances, such as with ultrasound images, meta-data (such as patient information) can be embedded within the pixel data. This is commonly referred to as “burned-in” metadata. While most image-based AI efforts currently use pixel data, there are efforts underway to process raw data, as it sometimes holds more information than pixel data[7].

Labels add further context, information, and value to image data. They can be study-level descriptors (e.g., this is an abdominal MRI) or image-level descriptors (e.g., on image 36, these pixels represent the liver). The radiology report that accompanies the images and indicates the findings, interpretation, and diagnosis that results from the images commonly serves as a source of labels. Labels can include:

- Radiology report findings, including Common Data Elements (CDEs)[20]
- Image annotations, such as arrows, measurements, and regions of interest on the images
- Extra labeling done specifically for data to be used for AI
- Non-image clinical data, including documentation from the electronic health record (EHR), pathology, laboratory, genomics, and other data
- Social media and other publicly available data, such as weather data and public maps
- Other data generated by patients, the public and the Internet of Things (IoT)

The performance of an image-based AI system depends on the diversity of the pixel data and the precision and accuracy of the labels. The radiology community can increase the quality of AI systems through standardization of annotations and measurements; traceability; data version control; documenting processes that alter, move, or store data; and correlation to patient outcomes and related meta-data[19].

Business operational and analytic data

Business operational data include data on customer transactions, employee tasks, resource utilization, and business processes. Information technology (IT) operational data include information on what, and how well, technology components are operating. Business/IT analytic data include data about speed and accuracy of IT processes, security and risk of the business-technological ecosystem, and measures of data integrity, validation, correlation, business efficiency, and productivity. Report turnaround time, relative value units (RVUs), scanner utilization, and quality measures are common examples of these data in clinical radiology.

Pre-training, synthetic, and augmented data

The performance of AI models improves as they are trained on more data. Excitement about the accuracy of AI models for perceptive tasks outside of medical imaging came from using datasets of millions or even tens of millions of images. By contrast, currently available radiology datasets for AI contain between hundreds to tens of thousands of radiology examinations. Thus, algorithms that drive radiology AI models are either typically pre-trained on large sets of non-medical image data, such as ImageNet (which has over 14 million labeled images of typical objects such as dogs, cars, and mountains), or use synthetic or augmented data[21, 22]. The process of applying models trained on one type of data to a different type of data is called transfer learning.

One approach to expand data for training is to use fully or partially artificial data, commonly referred to as synthetic data. Synthetic data are generated at least in part by statistical programs to randomize their features. Once the model to produce them is developed, generating synthetic data is fast and inexpensive. Synthetic data are useful for pre-training[23]. Risk of potential compromise of patient data with them is minimized, since the data are not obtained from real patients. For radiology, synthetic data can mimic rare diseases, allowing the algorithms to train on more exams showing the pathology when such exams are hard to obtain from patients. They are also useful for researchers, when no data exist, or to generate data to test and verify AI products.

Synthetic data are often used as adversarial images in adversarial networks (GANs), a class of AI algorithms[24]. While these images appear to simulate pathology precisely and can increase the overall accuracy of the trained model, there is little research or understanding of their effect on real-life settings. Synthesized models of pathology may perpetuate imperfect understanding of pathology and may be inaccurate. Also, because AI models can potentially pick up subtle features, synthesized images can introduce artifacts imperceptible to humans that may affect AI model training in ways we may not be able to appreciate. Until more is known about effects

of using synthetic data for training, anyone and any vendor using GAN-generated images for data augmentation needs to disclose their use.

Augmented image data are real data that are copied, with each copy altered in some way to make it different[25]. Common augmentations include rotation, flipping, translation, resizing, adding noise, or sharpening. Augmented data are useful when the algorithm to be trained can identify the object despite such changes. Often, augmented data are easier to generate than synthetic data, though augmented data may still have privacy and data use restrictions. Data augmentation techniques and use require caution. What appears to be a benign method of rotating images can have unintended negative consequences if not thought out carefully. For example, a patient with pneumoperitoneum on an upright radiograph, if rotated, can give false data to the training process because a decubitus radiograph of pneumoperitoneum appears quite different than an upright image turned 90 degrees. Details of any data augmentation used during algorithm training should be made available to users.

Synthetic and augmented data help fill in gaps in real data and are useful to improve reporting and selection biases. They may also exaggerate bias[26] however, if they duplicate or reinforce a systemic bias in the baseline data used to generate them. While these data are useful to train algorithms, much more research is needed to understand the ramifications and limits of using large amounts of artificial data in radiology and the criteria for their use.

Raw image data

Raw data are usually proprietary to companies that build imaging equipment, such as CT scanners. They are largely uninterpretable by humans. When digital radiology first appeared, digital data storage was expensive. As such, only data in forms thought to be clinically useful were saved, and the raw data was rarely saved for more than a short period after images were acquired and interpreted. Theoretically, AI can find features in raw data more robustly than from data that have been processed into human-interpretable images. Because of this, the radiology community is increasingly recognizing the value of raw data. Patients, industry, and researchers will benefit if raw image data are saved and made accessible in addition to traditional, post-processed image data[19].

Data ownership

Health care entities collect and protect patients' medical images and associated health information. Now, with robust methods to share data electronically and the need to aggregate data for AI, medical imaging data are increasingly being shared among radiologists, other health care workers, institutions, and even countries. Ethical and technical issues to secure data are complicated, especially as ethical norms and laws vary among countries. This complexity and

variation hinder sharing of patient data for clinical care, AI research, and commercial development.

On the surface, "Who owns patient data?" is a concept that radiologists, the greater medical community, and regulatory bodies have already addressed. Data ownership varies among countries. In the U.S., the entity that performs the imaging becomes the owner, though patients have a legal right to a copy of the imaging data. While practices are heterogeneous, many hospitals include permission to use data retrospectively for research in their general consent to treatment, which has been shown to be accepted by patients[27]. In the U.S., federal law does not require consent for de-identified retrospective studies as defined in the following excerpt from 45 CFR 46 (2018 version)

> (ii) Information, which may include information about biospecimens, is recorded by the investigator in such a manner that the identity of the human subjects cannot readily be ascertained directly or through identifiers linked to the subjects, the investigator does not contact the subjects, and the investigator will not re-identify subjects[18]

By comparison, in the EU, the General Data Protection Regulation (GDPR) specifically states that patients own and control their sensitive, personal, and/or identifiable data (both medical and non-medical). The GDPR requires explicit patient consent to reuse or share data, and patients may withdraw their consent at any time[16]. Each EU country has a national body responsible for protecting personal data[28]. A new EU-based initiative is actively asking patients to donate their data after undergoing an imaging exam and securing a diagnosis[29]. Sites where radiology examinations are performed are also subject to ownership and copyright regulation, suggesting that approval to use radiology data will require approval by both patients and imaging facilities.

In Canada, similar to the U.S., health care providers that produce medical images own the physical record, and patients have a right to access it [30]. Health care delivery is under provincial rather than federal jurisdiction, and varies between Canadian provinces[31, 32]. The recent Tri-Council Policy Statement: Ethical Conduct for Research Involving Humans[33] states that "consent is not required for research that relies exclusively on secondary use of non-identifiable information," a position held by Canada's largest research agencies.

While legal discussions on data privacy and ownership are outside the purview of this statement, they illustrate the need for new discussions on who owns what data; and if data are transferred, used and reused, who pays whom for what. In other words, might the owner of the imaging machine own the pixel data, while the radiologists own the labels they generate, including reports, annotations, or other information they contribute to the value of an exam? Until recently, most medical image data sharing and aggregation was for research purposes,

and governed by mature policies. As medical image data become commercially traded entities, if the their value comes from having both pixels and labels and that bundle is significantly more valuable than either part separately, who receives that value is yet to be determined.

Data sharing and data use

From search engines to word processors to digital assistants, the dislocation of data value has disrupted the business model. Traditional products are built less to provide services and rather as portals to collect, capitalize on, and profit from data. This paradigm has the potential to occur in medicine and radiology.

As medical data become more valuable, the line between academic and commercial uses of data is blurring. For example, suppose a hospital sells exclusive rights to their imaging data to a company hoping to build an AI product. Since patients also retain the right to access their data, may they, in turn, sell their data to another company that wants to build an AI product? Or may they refuse to share their data for commercial development but allow it for non-profit research? Many governmental and other funding sources now require applicants to share their data, so how will this be reconciled with exclusive data use agreements? Legislators and regulators need to revisit the policies that concern the use of medical data in academic and commercial settings, finding an equitable balance between the interests of society at large and the interests of the individual patients who generate the data[34].

The skyrocketing value of radiology data is disrupting traditional data-sharing practices, and buying and selling of radiology data is becoming more common. The data are most valuable to those who can best monetize it, and there is reason to suspect that the people who are best able to monetize data will be those who are least morally scrupulous. Current examples of questionable data use in social media and other settings reinforce this suspicion. A model of self-governance, by those who own the data, is unwise. So long as radiology data are held privately, we will need regulators with the power to ensure that the outputs of those who own the data — not just algorithms and clinical products — properly take into account the associated moral risks.

New deals for commerce in medical data may be influenced by naiveté or greed. For example, in 2015, the Royal Free National Health Service (NHS) Foundation Trust signed an agreement with DeepMind Health, giving the company access to 1.6 million personal identifiable records at no charge. It was suggested later that the NHS was “seduced by the magic of the algorithm company and in future should at least seek more control over the data and their transparency. What [the NHS] did not realize is that they were the ones with the really important thing, which is the dataset.”[35] In general, people don't conceptualize their data as being valuable, and undervalue it. A necessary condition on consent is being informed. A patient can be taken to

have consented to let others use their data only if they have been informed of how valuable those data are in monetary terms.

Increasingly, companies anxious to obtain data are attempting to contract with people outside radiology, including administrators, and clinicians and researchers in other specialties. For example, they might approach a pulmonary or surgical research group to obtain images to develop products for those fields. Sometimes these contracts are discovered by the radiology department only in retrospect, and the contracts include access to data in perpetuity or for exclusive use. Thus education on ethical access to data, including radiology images, as well as internal regulations and enterprise policy for data access, should be promptly established and publicized to the entire medical enterprise.

Social media demonstrates that substantial data value comes from its use in surveillance to build a deep and unfeeling profile of each person. Medical data is extremely valuable in this paradigm. Today we use robust "free" tools for internet search, email, document management, and social media image sharing in exchange for giving our data to companies that do with it whatever makes them money. Using this same business model, companies could potentially provide excellent "free" radiology AI tools, where the cost to use them is giving our medical data to them to use as they please. For example, one company today offers a free tool which enables people with little programming ability to build AI models[36].

Open, freely accessible radiology data offer benefits for the greater good of patients, society, and the economy. Several U.S. universities recently publicly released moderate to large datasets[37, 38]. During the 2018 annual meeting of the French Radiological Society (SFR), the foundation of an AI ecosystem (DRIM France IA) was announced. The idea is to build a qualified database of more than 100 million medical images within a period of 5 years, which can be used by companies willing to develop AI tools that will be made freely available to France's hospitals and radiologists. At the least, countries should develop a consensus regarding what sorts of data sharing is legitimate, and develop guidelines on how data producers, owners, managers, and users may share data safely and equitably. Despite such efforts, it may be naive to expect most data owners to give away valuable resources for free.

Release of information and data use agreements (DUA) are critical tools to ensure that data are used transparently and ethically. DUAs explicitly specify what the involved parties can and cannot do with a dataset, and how they must dispose of the data once the agreement ends. This is complicated, however, by the need for version control of data used to train, test, and validate algorithms. Should those data be saved and appropriately documented for the life of the algorithm, or possibly for some period related to the effect on a patient of any decision the AI product made? If the data are used for continued learning and downstream algorithm

descendants of the original or parent source, should the data be version controlled for that lifetime as well?

DUAs must be updated regularly to reflect new uses of patient data. Data may be considered entities unto themselves. Data flexibility influences their value. The more they can be repurposed, combined, and shared, the more valuable they become. As these changes occur, each data state should be documented. DUAs may include limitations on certain instances of reuse to avoid breaches of privacy and biases in training algorithms. Subsequent DUAs need to include version control specifications, particularly when data are used to train, test or validate AI models. They will include new and more comprehensive rules for data reuse and intellectual property. The entities receiving the data should take responsibility to identify the origins of those data and fully understand the permissions and rules attached to them. It has been suggested that each patient sign a DUA with any third-party entity that contributes to their digital health record, to encode data quality, security and use for all contributors and users[39]. Another approach is dynamic consent, an electronic process which allows ongoing communication between researchers and research participants[40].

We specifically note DUAs that include exclusive use of data as unethical, because such agreements may remove a significant amount of useful radiology data from general use. They can exacerbate concentration of power and erode transparency. We should strive to make radiology data widely accessible, both legally and financially. This means that we should curtail exclusive data access contracts and that we should try to ensure that datasets — even those that have had substantial work done to increase their value, such as linking, cleaning, and de-identifying, or being coupled with high-value labels — are capable of being accessed by entities with lesser financial resources.

Institutional review board (IRB) requirements also need to reflect new uses for patient data. Some IRBs, particularly outside the U.S., waive consent requirements when they are not feasible or impede validation of a research study or AI model. When might patient privacy and consent not be absolute, and patient's interests be overridden, when risks are low and there is a compelling public interest to use the data for the greater good[41]? If this occurs, patients should be made aware.

The need for a robust technical infrastructure to share and manage medical data is driving new supporting technology. Federated learning is an approach gaining wide favor, where a supervised learning algorithm is delivered to a health care institution which allows the data to stay inside the institution's firewall[42]. This is probably the best way for an imaging site to control its own data. This approach requires each individual health care institution to have its

own algorithm-hosting infrastructure, and to prepare and label their data in a manner that the algorithm can accept.

Blockchain models theoretically provide a strong, comprehensive method for individuals and entities to securely aggregate and easily access medical data across disparate sites[43, 44]. Details and issues of this technology are outside the scope of this Statement.

In the interest of full transparency and trust, it would be beneficial to provide a framework to recognize the value of patient data and provide guidelines for different use cases. What must radiology do to gain patients' trust that their data are being used appropriately? How should radiology help patients understand if they have any claim on the monetary or other value of their data? Claims on monetary value are based more on legal precedent than ethics, and vary by country. Most patients are willing to have their data shared[45], and presumably trust it will be used appropriately. The purpose of data sharing, such as for research versus commercial product development, changes patients' willingness to share data[46]. This may not hold in the future, however, if breaches in research data compromise patient privacy or as patients realize the monetary value of their data[47]. This is a complex setting. Suppose a patient withdraws consent upon learning a research project in which they participated is now being commercialized. However, the FDA submission has already been completed, so now should the model be retrained without this patient's data? This will necessitate a new submission to the FDA under current guidelines. Thus, organizations may need to be more forthcoming with the possibility of commercial product development from research activities in the informed consent process.

Increasingly, individual patient data are being collected outside of formal health care settings. Patients and the public may be invited to share[29, 48], or even sell, their radiology examinations. Today there is no consensus on consent agreements or contracting rules for how these data may be used and reused, nor are there requirements to notify patients how their data are being used, or by whom or for patients to notify anyone about selling their data outside of health care settings. It may be possible for a patient to sell the same data to multiple parties, and thus contaminate test and validation datasets, or adversely introduce bias in training.

Patients have large amounts of easily identifiable data outside of radiology. These include other medical data from their health record, pathology and genomics, data from mobile phones and personal health and exercise tracking devices, internet search history, socioeconomic data, location tracking, video cameras, and environmental data such as weather records. These data, many of which are publicly available, can theoretically be aggregated to provide broad and deep "360-degree" views of patients. These integrated data may enable more accurate diagnosis and treatment options for individuals, but they are nearly impossible to de-identify

and carry significant privacy risks. This is even easier when a patient has a rare or unusual disease.

Patients seldom know where their data go. An important way to establish trust is through transparency. Making patients fully aware of an entity's data practices, and ensuring that they can learn about, participate in, and in some cases even dictate the collection and use of their data, builds customer confidence and has the added benefit of greater brand loyalty. Doing this will also require the entity to understand its goals for sharing or reusing data. Some of this relies on context. If patients find their data used in a context where they do not expect to find it, the patient's surprise can quickly change to mistrust.

Data privacy

The right to privacy has been defined as the right "to be let alone," and to be free of surveillance by other people or entities[49]. In this setting, only authorized individuals should have access to patient data. All reasonable efforts should be made to preserve this privacy, particularly as data are reused and move through chains of ownership and responsibility.

In the U.S., the Health Insurance Portability and Accountability Act (HIPAA) defines strict privacy policies for patient identifiers considered protected health information (PHI). Because of this, data often are de-identified or anonymized, which obscures or removes identifiers from health information before being used for research or commerce[50]. Medical images pose unique de-identification issues. For example, images of the head and neck can be reconstructed into 3D models of patients that can be fed into facial recognition software[51]. Radiographs may incidentally include identifying information on bracelets or necklaces, or serial numbers on implanted devices such as pacemakers or defibrillators[52]. Ultrasounds may have identifying information burned into the image pixels. Radiology images also include extensive metadata, some of which identify the patient. Private DICOM tags, used in a proprietary fashion by vendors and therefore frequently undocumented, may unexpectedly hold information that identifies patients, institutions, or a patient's disease.

When one uses these data to extract features and train AI algorithms, the model may train on these data, and then may not work when those data are unavailable in other settings. De-identification of radiology examinations requires additional steps beyond deletion and replacement of the content of DICOM tags, and may necessitate manual review of images by humans. Some academic centers in the U.S. prohibit public sharing of data until two individuals have manually reviewed and cleared each item to be shared.

Despite de-identifying radiology exams and other medical data by rigorous traditional means, these practices are not absolute. Using a 360-degree approach described previously, entities

with expertise in manipulating massive data can likely re-identify just about any radiology exam[53]. It is technically feasible for a large social media company to gather data from smartphones and personal devices, along with online search history, and purchase and match these with health care data. They could then advertise to those individuals, or sell those data to insurance companies, hospitals, nursing homes and others. Radiology groups might find those data valuable to identify patients who need future imaging. This sort of all-encompassing information access further underlines the need for, and importance of, data security. Bad actors with access to medical data could extort patients about aspects of their medical history that they wish to remain private.

Ethical practitioners will make data as private and secure as possible, while also being transparent that medical data may not ever be absolutely private. Perfect anonymization is challenging at best.

Data used to train algorithms presents another new setting for data exposure. Commonly used deep-learning approaches often incorporate details about the training data. The algorithm's behavior may inadvertently disclose these elements [54]. More nefariously, algorithms can be intentionally designed to leak sensitive data, a process known as intentionally back-dooring[55]. Thus, AI deployments may need additional precautions in addition to normal institutional software acquisition security policies.

Bias and data

Bias is a systematic deviation from the truth. Bias caused by data occurs when the sampled data do not represent the truth. This is complicated because different settings may have their own truth, such as "truth" about one demographic group may not accurately represent truth of a different group, or in a different setting. Types of bias most common in radiology AI include reporting, selection, and automation. Automation bias will be discussed in the Ethics of Practice section.

Reporting bias is when the reported, or presented, data do not completely represent the real world because data are selectively disclosed. In medicine, this may come from clinical data being more available for positive research findings, or from those same data being duplicated or over reported. On the other hand, data from negative studies are often under-reported. It also occurs when prototypical data are assumed, for example, describing bananas without noting their color as yellow, because it is assumed bananas are yellow unless otherwise noted[56].

Selection bias or sampling bias occurs when the sample does not represent the population accurately[57]. Often this is a result of using available or interesting data. Using data from one

institution to train an AI model, for example, may accurately represent the population of that institution, but not the more general population for which the model is intended. It may inadvertently discriminate against underrepresented subsets of the population[58].

Selection bias may occur overtly or inadvertently. For example, if all the images for a radiology AI algorithm on a particular disease come from a cohort based on a set of features different from what represents the entire population on which the algorithm will be used, it may systematically give the incorrect answer for individuals who do not match the training group's features. Depending on the question to be answered, relevant features range from physical and health characteristics such as age, sex, sexual orientation, weight, height, race, and genetic and medical history to economic, ethnic, and educational features. Because AI often utilizes larger amounts of data and extracts features at a more granular level than humans, it is often difficult to know in advance which features of a training group may bias or otherwise result in a clinically unethical AI model.

Dataset shift (DS), a subset of selection bias, is a significant barrier to widespread AI use today. DS exists in most radiology settings because image data used for training do not accurately reproduce the conditions of future imaging studies. This includes bias introduced by experimental design, such as the use of synthetic or augmented data. In other words, previous exposure to training is inadequate for the model to make accurate predictions in new situations[59]. While radiologists commonly notice and adapt to differences in images due to slice thickness, scanner brand, field strength, gradient strength, or contrast timing without affecting image interpretation, AI generally lacks that ability. For example, if an AI agent is trained only on images from a 3 Tesla MRI, it may or may not generate the same results on examinations performed at 1.5 Tesla. Similar situations exist for each of the parameters above. One approach to mitigate DS is to have comprehensive training, validation, and test sets representing every type of image data acquisition and reconstruction[60, 61]. A second solution is to develop mathematical processes to recognize, normalize, and transform data to minimize DS.

In countries with few radiologists, applying AI trained on datasets from wealthy countries represents unique DS risks. For example, could an open source chest X-ray algorithm developed in Southern California produce harm during a SARS outbreak in rural Asia or Ebola outbreak in Africa?

Some types of dataset bias occur commonly enough that algorithms can distinguish between different datasets. Manually selected data fundamentally include more bias than data chosen randomly or automatically. Curation bias may occur when humans can choose from which angles to take images, which commonly occurs in ultrasound. Negative set bias arises when datasets over-represent positive or otherwise interesting examinations. This is particularly

complex for radiology, where the vast majority of exams are normal. One then needs to balance collecting enough examples of pathology without aberrantly biasing the algorithm. When synthetic or augmented data are used to generate enough examples of rare pathology, they may inappropriately bias the dataset.

Radiology data are often unbalanced, meaning they have many cases of some categories, particularly normal examinations, and few cases of pathology. In unbalanced datasets, categories may be undersampled or oversampled to improve model performance or runtime. This may introduce bias.

Bias is sometimes thought of as ethically neutral, as a tendency to produce differential outcomes. In this scenario, bias could be beneficial. If health systems currently deliver subpar care to certain subpopulations disproportionately, there may be an opportunity to rectify that inequity using AI tools that prioritize good health outcomes for all patients or subpopulations. We believe, however, that it is best to think of bias as a negative thing, and the ethical approach in radiology AI is to minimize bias.

Data labeling and ground truth

AI models in clinical radiology today use supervised ML, where the model learns to match given labels to given images well enough that when the model sees new images, it accurately predicts what label to match to the new images. This is most useful when labels match ground truth, which is the truth about the state of the patient and the patient's pathology or lack thereof.

Defining ground truth in medical imaging is problematic. For example, an AI model could be trained to recognize a fracture of the scaphoid bone in the wrist. The ground truth labels to train the AI model may come from a radiologist labeling the images as yes or no for fracture. Some fractures are too subtle to see on the initial examination, or the fracture might be visible but missed by the radiologist. For the clinical setting of a question of fracture of the scaphoid (a small but significant bone in the wrist), if the initial X-ray is read as normal and the patient still has pain two weeks later, the exam is repeated to look for a fracture which may have been occult initially but typically easier to detect on the later exam. Would the initial report be accepted as ground truth, or in this case would ground truth include a check to see if repeat X-rays were done later, and what they showed? In other words, what clinical outcome is most important? For some radiology examinations, the ground truth label will come not from a radiology report, but rather from a combination of subsequent imaging, physical exam findings, surgical outcomes, pathology results, genetic analysis, and other clinical data.

Not only will a radiologist fail to label 100 percent of examinations correctly, they may label exams differently the next day, or from another radiologist. Ground truth using qualitative

scoring by a single expert may be confounded due to this intra- and inter-observer variability. Interpretation by more than one radiologist improves label accuracy[62]. If three radiologists were to evaluate each examination, one could formulate ground truth from their majority or consensus interpretation; in practice, this is prohibitively expensive.

Alternatively, semi-quantitative scoring systems can be developed to determine an imaging ground truth, with rigorous rules set out in scoring atlases and with assessments performed by multiple readers. Formal techniques to evaluate image-based scoring systems such as these include the OMERACT Filter[63]**.** An AI system might be deemed successful if it performs at least as well as other human expert readers at one of these scoring tasks. For the scaphoid fracture, a semi-quantitative grading system might assign a score based on features such as cortical interruption, presence of lucent line, change in bone density, and how the other wrist bones are aligned.

This illustrates the multiple challenges in defining the ground truth labeled data to train AI algorithms. What should it be based on, and who should determine that? To avoid deep-seated biases, the answers will depend on the specific task, and need to be carefully considered and defined *a priori*.

An ethical approach suggests one should weigh the need for improved ground truth labels against the feasibility and cost, and provide transparency about how ground truth is determined for each dataset. This suggests that radiology and medicine would be well-served by standards for discovery and reporting of dataset bias. The radiology community should ask questions about their data, and be transparent about the data evaluation process and the answers to these questions. This is particularly important when using publicly available datasets for training, as researchers may be unaware of assumptions or hidden bias within the data.

When an AI model is implemented, those responsible should be able to answer these questions, and other similar questions, about the Ethics of Data:

- How will we document and notify patients about how data are used, both by us and others?
- How do we document data used to train an algorithm, including descriptors for features traditionally associated with bias and discrimination.
- How and by whom are labels generated? What bias might arise from the processes used?
- What kinds of bias may exist in the data used to train and test algorithms?
- What have we done to evaluate how our data are biased, and how it may affect our model?

- What are the possible risks that might arise from biases in our data, what steps have we taken to mitigate these biases, and how should users take remaining biases into account?
- Is our method of ground truth labeling appropriate to the clinical use case we are trying to resolve?

Ethics of Algorithms and Trained Models

At its core, AI employs classification systems to come to a result. The first and perhaps simplest approach to AI is formal logic: “If an otherwise healthy patient has a fever, then they may have an infection.” A second approach is probabilistic, or Bayesian, inference: “If the patient has a fever, adjust the probability they have an infection to X%.” A third approach generalizes from similarities to make new predictions: “After analyzing the records of patients whose temperature, symptoms, age, and other factors mostly match the current patient, X% of those patients had an infection.” A fourth approach, neural networks, mirrors the function of a neuron): “If enough signs and symptoms match a specific pattern of previously labeled data within a model, then classify as diagnosis X.”

Machines making decisions

Decision-making is the selection of a belief or a course of action among multiple alternatives. The decision may trigger an action. Human decision-making is the process of choosing alternatives based on the person’s knowledge, values, preferences, and beliefs. AI agents choose alternatives based on features in the input data. For supervised learning, the algorithm chooses that alternative based on prior training to match data features to labels. It is within the labels where human values, preferences, and beliefs may be transferred to the model. This is where human bias may manifest.

While AI performs well with classification tasks, it is a machine, not a human, and does not calculate fairness or equality[12]. Fair is not an AI concept. Responsibility for these concepts falls to humans, who must anticipate how rapidly changing AI models may perform incorrectly or be misused, and to protect against these possible outcomes, ideally before they occur[64].

AI models consist of the algorithm and the data on which they were trained. To reconstruct algorithm development and testing requires saving, or having the ability to reconstitute, exact versions of the datasets used. In theory, AI models can be built to change continuously based on learning from new data. Current AI models are trained on a carefully crafted dataset, and then halted while used clinically. If the model is responsible for a high-risk decision, it is unclear if incremental benefits from continuous training will outweigh the risk of unintended

performance declines. This version control process of freezing and documenting each working version of a model is standard practice, but until now such rigor has not applied to data associated with producing an AI model. Similarly, other common software quality control policies and best practices for ethical software management may now apply to data. This is a critical issue, as it will be almost impossible to find root cause and provide corrective action for performance failures without knowledge of exact data used. This has important implications for both federated learning and transfer learning, not only due to issues of data accounting, but also because the regulatory framework may prohibit postmarket model improvements or model training on private data.

Radiology should start to prepare for the following type of scenario. Suppose Hospital A decides to purchase an FDA-cleared lung cancer AI model from vendor ABC that has a very high published accuracy. However, when installed, Hospital A obtains much less accuracy using its own data, and wishes to retrain using those data after purchasing the model. Should Hospital A be allowed to do this? Should the vendor allow it? Should the vendor have the option not to allow retraining? Is the vendor liable for this modified AI model or does this void any warranty? Suppose the vendor allows sites to retrain on their own data. Thus, multiple hospitals might then have unique versions of the software. Is each hospital responsible for their own version control? What happens when the vendor releases a new version? We may need a mechanism with standard infrastructure and documentation methods to maintain version control not only of the vendor's parent product but of all descendant models, whether from the vendor or those modified locally.

For the foreseeable future, radiology AI will be based on well-curated datasets and code freezes. AI is theoretically best when allowed to learn continuously. At some point in the future as we gain more experience with how AI models fail, and how to monitor them, new processes and regulations will arise which enable continuous learning.

Algorithm selection

The first steps of developing any AI solution are: understanding the training data, defining model assumptions, and critically evaluating for bias. Choosing an algorithm depends on the size, quality, and nature of the data, available computational time, and the task to be performed. Some algorithms work better with smaller sample sets, while others require numerous examples. For image recognition purposes, convolutional neural networks (CNN) have shown some of the most promising results. Developers select algorithm structures (e.g., linear vs. non-linear) based on assumptions or analysis of the training data. Ethical issues, beyond understanding which algorithm type best suits the situation, include consideration of what algorithm might give the most useful output for patient care, balanced against limited computing resources or the amount and type of training data available.

The objective of a model can also introduce bias. When selecting trained models, radiologists should consider possible unintended consequences, and evaluate the fairness of the model's performance across the real-world data of multiple patient groups. This is best done by ensuring that data the model will analyze in practice matches the training and test data used to validate the model's performance. This process is like applying evidence-based medicine principles when considering the results of a diagnostic test or choosing a treatment.

Due to lack of adequate personnel to develop and train AI algorithms and increasing algorithm complexity, a new field of automated ML algorithms is developing. These allow domain experts with limited technical computer science skills, such as practicing radiologists, to build and train AI. While this has potential to improve democratization of AI, unskilled trainers may be unaware of complexity and potential pitfalls of AI models. As radiologists become increasingly responsible for creating and supervising AI, they should learn enough to understand not only how to optimize algorithms, but also the ways in which those algorithms may be unethical, biased, or otherwise not work as intended. This topic requires complex mathematics and statistics which in general is outside of radiologists' knowledge. They should acknowledge this and involve appropriate experts.

This review is largely focused on image analysis with neural networks and deep learning. Many other types of machine learning algorithms are available, which may be appropriate in different situations, and entirely new classes of algorithms are being developed. Some of those may soon displace deep learning for image analysis.

Algorithm training

Once an algorithm has been trained on a dataset, it is known as an ML model. This step by itself may introduce bias, as the algorithm inherits decisions made from data selection and preparation. To minimize bias (particularly dataset shift) and maximize benefits for patients, it is critically important to train models with datasets that truly represent data the model will see when it is installed in multiple disparate radiology practices. Often this requires training across multiple institutions and diverse datasets. One helpful approach is the previously described Federated learning method to share models between institutions, including their weights and parameters. This may be a good option since models are not governed by patient privacy regulations and data can remain inside an enterprise's firewall.

Model evaluation and testing

Once the model is trained, it is tested with different data to see how well it works, and potentially how it handles atypical input data or data that it would not be expected to process well. Model testing includes selecting the right test data, defining metrics to evaluate model

results, and determining who performs testing. Model evaluation may include both a validation phase and a testing phase. During validation, data different from the training set are repeatedly shown to the model and it is refined. However, the eventual testing phase should present a third, separate dataset to which the model has not been previously exposed, and it is the model's performance on this dataset that should be reported.

For any supervised technique, the choice of ground truth against which the AI model is to be evaluated must be selected, potentially including imaging features and/or outcomes as discussed above in Ethics of Data. Even after ground truth has been selected, ethical difficulties arise. For example, when faced with clinical situations where there is a high level of uncertainty, humans tend to err on the side of caution, evidenced in a study in which it was difficult to separate benign and malignant skin lesions, with doctors over-diagnosed malignancy[65].

During the testing process, data should be checked to ensure it matches the deployment context. It may be necessary to perform baseline statistics on the training and testing data to understand disease distribution. The confusion matrix defined as (TN + TP + FP + FN) is commonly used for binary classification problems (Figure 1).

		**Prediction**	
		Yes	No
**Truth**	Yes	TP	FN
	No	FP	TN

Figure 1. *Confusion matrix showing the instances in a predicted class versus instances in the actual class. From this table, it is easy to see how often classes are mislabeled. TP=true positives, TN=true negatives, FP=false positives, and FN=false negatives.*

For thorough testing, different classes/groups should be assessed to model performance. For example, there should be a confusion matrix for the general population, one for females, another for males, and so on — to ensure that any gender bias shows. The testing dataset for the model should have demographic parity, where every test subject has an equal chance of being selected. It should also have predictive parity, where subjects' predictions have an equal chance of a positive predictive value truly belonging to the positive class[66]. In practice, it may be difficult to get a balance of all four components of a confusion matrix. Hence, other elements of the confusion matrix, like the false positive and false negative rate balance, should be

considered. New metrics like equalized odds allow model testing to satisfy the false positive and false negative rates.

Radiologists faced with a diagnostic dilemma commonly understand the cost of under- and over-diagnosis, and weigh these factors in their decision-making. For instance, a radiologist reading a chest radiograph with equivocal findings for abdominal free-air will sacrifice specificity due to the clinical consequences of missing pneumoperitoneum. While impacts such as adverse events or social factors are not easy to model or assess, ethical algorithm creators should strive to measure algorithm performance in true application beyond simple accuracy. Often this will require more sophisticated statistical analysis than the typical area under the curve (AUC) calculations derived from the TP, TN, FP and FN.

In light of the known legal, privacy, financial and other resource challenges of access to data, developers may opt for the minimum model training required for FDA certification. The relationship between a legally certified model and a model that functions robustly, correctly, and ethically in the wild is still to be defined. It may well be that, at least to start, legal certification may not equate to a radiology AI model being safe or clinically useful.

Beyond technical testing and validation, models will need clinical validation. How do they work in production, on real, new, patients? In general, models provide discrete predictions, while patients are distributed across a continuum. Models will need to show they are clinically useful and clinically ethical when confronted with real people the model has not seen previously.

Transparency, interpretability, and explainability

Transparency, interpretability, and explainability are necessary to build patient and provider trust. When errors happen, we investigate the root cause and design systems to eliminate the potential for similar errors in the future. Similarly, if an algorithm fails or contributes to an adverse clinical event, one needs to be able to understand why it produced the result that it did, and how it reached a decision.

Some types of AI commonly used in radiology, such as artificial neural networks, are “black boxes,” and historically it has been problematic to understand why they make specific decisions. This black box approach is problematic for patient care, where decisions potentially have high consequences. It must be acknowledged that the workings of the human mind also represent a “black box”, to some extent. Nonetheless, a human radiologist will usually be able to explain a line of thought that led to a conclusion. A similar level of traceability is also necessary to ensure confidence in AI-based decisions. It is always important to note that an AI product is not human; it is a computer program envisioned, built, and monitored by humans.

Interpretability is the ability to understand the workings of an AI model. Explainability is the ability to explain, in terms that a person understands, what happened when the model made a decision. Explainability includes understanding why a model made a particular decision, or appreciating conditions where the model succeeds and where it fails. Explainability includes both comprehending technical aspects of algorithm structure and how outputs are presented to the user [67]. In complex networked systems of AI models, users may be other AI models further downstream in a cascade of decision-making machines. Explainable AI (XAI) has been recognized as a core area of research, with funding opportunities from agencies such as the Defense Advanced Research Projects Agency (DARPA[68].

For a model to be transparent, it should be both visible and comprehensible to outside viewers. How transparent a model should be is debatable. Transparency might make it more susceptible to malicious attacks, or reveal proprietary intellectual property. Furthermore, imposing a wide definition of transparency could jeopardize privacy by revealing personal data hidden in underlying data sets. In general terms, the more transparent AI is required to be, the less complex it can be. This may impose limits on its performance[69].

Even if we can "look under the hood," the ML process often is extremely complex, with up to billions of parameters and complex mathematical operations. Pinpointing a causative bug in such a system is a daunting task[70]. A more practical approach may be to advocate for visualization and explainability.

The GDPR states that automated decision-making systems that have significant impact on a person are not permitted without that person's consent[16, 71]. It also states that the individual has the right to an explanation of how the automated decision was arrived at, and the consequence of that decision[72]. This has been interpreted to mean that AI decisions should be able to be rationalized in human-understandable terms[73]. However, this "right to explanation" is, of necessity, limited. The European Council Data Protection Working Party interprets this as conferring a right to the *envisaged consequences* of a process, rather than an explanation of a particular decision[74].

The radiology community should create guidelines for explaining as well as assessing AI models. These guidelines will need to consider the variety of clinical applications. For example, AI built into an MRI scanner to decrease scanning times will have different impacts on patients, and potentially different technical pitfalls, than image analysis algorithms. Considering the GDPR definition, is decreasing scan time a decision that has a "significant impact" requiring patient consent? Does every image analysis AI decision have a significant impact?

It is unclear how much of an AI solution's inner workings radiologists have a duty to assess before applying the AI in patient care, and just how transparent AI vendors should be regarding the inner workings of their product. May a vendor supply a canned explanation of what its AI models do, or does each radiologist need intimate knowledge of the model and the ability to explain it clearly to the patient? What represents an adequate explanation?

In many instances, where AI is used to augment medical decision-making, and a human physician has final authority, there will be no legal requirement to explicitly inform patients of the use of AI in their care. Conversely, where AI represents the principal point of contact between a patient and health care (e.g. AI tools directly offering advice, or triaging patients for care), patients should be clearly made aware they are dealing with an AI tool[69].

Open source software

To verify published research on radiology AI requires access to the algorithms discussed. This open source software (OSS) approach has been used for other fields and software. OSS has its own ethical issues, outside the scope of this statement. This includes resource consolidation, potentially biased and exclusionary groups producing it, and internally code-focused approaches[75, 76]. Strengths of the OSS approach include transparency, access to code, and potentially more robust and secure code.

Replicability

AI models should be replicable; the model should give the same or better result if given the same input. While this seems obvious, it is in contrast to humans, who commonly exhibit both inter- and intra-observer variability. The standard for an ML model should at a minimum match expert human performance. Replicability is problem-dependent, and the amount of variability depends on the specific task at hand.

Algorithm bias

Computer-assisted decisions are dependent on the quality and accuracy of the data upon which they are derived. As described in detail above, any bias in the data will have an impact on the outcome, much the same way that humans can only base decisions on their own previous learning.

Implementing ethics of AI within medical imaging is dependent on the continuous verification of both the data and models. Deployed models will need to be monitored and re-tuned if a source of bias or new information are identified. There is an opportunity to invite diverse stakeholders to audit the models for bias. Mechanisms should be put in place to monitor user

reports and user complaints. Before model deployment, training data should be matched with deployment data. The metrics for performance should be thoroughly tested and used to inform real-life performance.

Security

Adversarial attacks are well-known in other AI domains, and the radiology AI community is becoming aware of them[77–80]. Currently, radiology as a field has no defense against such attacks. While potential solutions may exist, this weakness must be acknowledged and addressed. It will become increasingly important for AI models to be incorruptible and robust against malicious manipulations and attacks.

When an AI model is implemented, those responsible for any part of its lifecycle should be able to answer these and other similar questions, about the Ethics of Algorithms:

- Are we able to explain how our AI makes predictions?
- How do we protect against malicious attacks on AI tools and/or data?
- How do we create sustainable version control for AI data, algorithms, models and vended products?
- How will we minimize the risk of patient harm from malicious attacks and privacy breaches?
- How will we evaluate trained models before clinical application, for clinical effectiveness, ethical behavior, and security?
- How will we monitor AI models in clinical workflow to ensure they perform as predicted and that performance doesn't degrade over time?

Ethics of Practice

Radiology AI is a complex ecosystem of clinical care, technological and mathematical advances, business and economics. Moral behavior, doing the right thing, can be intellectually uncertain. We see daily how technical innovation crosses into unprincipled activities, and even if unintentional may cause considerable harm to patients, society, and our own reputations. Conscientious ethical values should guide decisions about where to apply AI, define metrics to describe appropriate and responsible AI, and recognize and alert the community to unethical AI.

At minimum, how do we evaluate and ensure that data obtained from patients and others is used in ways that benefit those from whom it is acquired? Do the data accurately reflect the appropriate cohort? Is the result of the AI fair? Does the result discriminate or harm anyone,

and if so, how and to whom is that made known? Do we share our technical insights with regulators, and inculcate ethical compliance into our practice and regulations? Are we able to explain how our AI makes predictions?

Radiology AI will exist in a much larger AI ecosystem. Changes to radiology may well change how a hospital is run, how hospitals are designed and built, and relationships between radiologists and patients, other physicians, administrators, IT staff, insurers and regulators. AI induced changes to the hospital and health care operations will also impact how radiology department and radiologists work. Radiologists with informatics expertise will be in high demand , and play key roles in radiology and hospital hierarchies. Ethical training must be prominent in the informatics radiologist's toolkit.

Many of the decisions that will be made about radiology AI will be made by others in business computer technology. Those people live in entirely different worlds from medical doctors. Business people are in the business of making money. Tech people are in the business of making machines better, faster, and easier to sell. Neither group are in the business of making patients better. For ethical radiology AI to succeed it must consider these goals.

Computer - human interaction: Keeping humans in the loop

The Institute of Electrical, and Electronics Engineers (IEEE) recently stated that autonomous and intelligent systems "should always be subordinate to human judgement and control,"[12] which in the radiology context will ultimately fall to radiologists. This is certainly one way to approach AI, though it fails to acknowledge the potential ability and significant benefits of autonomous AI tools.

The doctor-patient relationship is predicated on trust. As medicine increases in complexity, trust extends from individual providers to larger health care institutions. As health care institutions and individual practitioners implement AI, maintaining transparency will be important to maintain trust[6].

It is ethical to be transparent with patients and all stakeholders when a decision is made by, or heavily influenced by, an algorithm. This raises intriguing issues about how to have a shared decision-making discussion with patients when AI is another party in decision-making.

Radiologists and institutions using AI in radiology should be transparent with patients about what is happening to them and their data. Patients should be made aware of:

- The ways in which humans oversee the decisions made by AI

- How AI is being used in diagnoses and medical recommendations, and what controls the institution has put in place to assess, validate, and monitor the AI tools being used.

Ethical oversight must extend beyond the end users of AI tools. Those responsible for developing, adapting and maintaining AI tools must also adhere to ethical principles[12]. Specifics of ethical behavior for those developing and maintaining AI tools may be different from those utilizing or implementing the tools. Equally, those whose interests are more-focused on economic gains from AI implementation such as practice managers and payers must be included in the ethical considerations and decision-making. Health care providers are already advertising perceived benefits of AI as a means of attracting patients. AI systems could very easily be programmed to guide users to clinical actions designed to meet quality metric requirements, or to increase profit, without necessarily conferring any benefit on patients. As complex dynamic networked systems evolve, it may be difficult to attribute responsibility among different AI agents, let alone between machines and humans[81]. Furthermore, the ethical principles required by those developing, adapting and maintaining AI tools may differ from the principles of those using or implementing the AI tools. Constant dialogue will be required between developers and users to ensure that both groups adhere to common standards of ethical behavior, and understand any differences that exist.

Many companies working in the area of AI have established ethics boards and adopted ethics charters. This is to be welcomed. It is vital that these bodies and their activities represent sincere efforts to maintain high ethical standards, and not “ethics washing”, designed as a strategy to avoid external regulation. In some instances, questions have been raised by outside observers about the transparency of these groups regarding their membership, recommendations and influence on commercial activity and decision-making[82]. Such ethical bodies should be truly independent of commercial influence to ensure trustworthiness.

How should oversight be maintained? Certainly there must be committees, boards, or working groups tasked with scrutinizing the introduction of AI, its clinical use, and outcomes from that use. The composition of these bodies should, to the extent possible, include all stakeholders involved in or impacted by the use of AI, especially including patient representatives. Individual radiologists, through continued medical education to improve their understanding of AI, can contribute by actively monitoring model performance as they use AI in their daily clinical practice. A mechanism to gather, compile, and disseminate information on the limitations, pitfalls, or failures of each AI model can help ensure transparency and continued quality assurance and improvement.

Tasks or decisions that should not be delegated to models need to be identified to ensure human oversight and prevent potential harm to patients. Whether these oversight bodies need

formal legislation to mandate and maintain them will be a matter for each jurisdiction. It may be sufficient for the authority of these bodies to rest within professional organizations, hospitals or academic health care structures (once these institutions are trusted by their staff, their patients, and the public). The legal question of treating autonomous AI agents differently from those under direct human supervision is under consideration, and yet to be decided[83].

Education

Rather than AI replacing radiologists, technologists, and other roles in radiology, new and different skills will be needed to practice AI-enabled radiology. This offers a unique opportunity to reassess the essential components of radiological work and determine the optimal combination of humans and AI to perform these tasks. Radiology needs research and specific guidance on training and protocols for both radiologists and patients for new, shared decision-making paradigms. Part of this training will need to focus on the practical question of how best to use the new AI tools that will be made available. But part of this training will need to focus on the ethical matters that arise by virtue of employing new AI tools. Beyond the details of ensuring ethical collection and use of data, and ethical development of algorithms (both of which processes will be driven and controlled by relatively small numbers of individuals), there are responsibilities to apply the algorithms in practical day-to-day patient care in an ethical fashion. These fall to every physician whose practice uses AI tools. The best way to ensure that AI tools are used ethically is to make the physicians who use them daily aware of the moral risks they face when using these tools. The better trained radiologists are, the fewer cases of wrongdoing there will be, blameless or otherwise.

Automation bias

Automation bias is the tendency for humans to favor machine-generated decisions, ignoring contrary data or conflicting human decisions. The literature contains several examples of automation bias that occur when humans monitor or observe decision-making machines particularly in highly complex situations [84]. Automation bias leads to misuse of decision-making machines, including over-reliance, lack of monitoring, and blind agreement[85]. Automation bias in clinical decision support systems has been well reviewed[86].

Automation bias leads to errors of omission and commission. Omission errors occur when a human fails to notice, or disregards, the failure of the AI tool. High decision flow rates, where decisions are swiftly made on radiology exams and the radiologist is reading examinations rapidly, predispose to omission errors. This is compounded by AI decisions made based on features that are too subtle for humans to detect. Commission errors occur when the radiologist erroneously accepts or implements a machine's decision in spite of other evidence to the contrary.

Radiology confronted automation bias years ago with the original use of computer-aided detection (CAD) algorithms in the interpretation of screening mammography. A few studies suggested that the original algorithm had reduced interpretation accuracy[87] and decreased sensitivity in a subset of radiologists[88]. It was theorized that reduced accuracy may have been related to over-reliance on CAD outputs. While today's AI-based CAD algorithms show much greater promise than traditional CAD in experimental settings, it is not clear how human-AI interactions will impact accuracy or efficacy in actual clinical settings. This will be partially addressed through validation processes like FDA approval, which will include evaluation of safety and efficacy. An element of "soft governance" is also useful; AI (or other products) are unlikely to be widely purchased if they cannot show compliance with accepted standards (whether required by legislation or not)[89].

There is a risk that resource poor populations may be harmed to a greater extent by automation bias because there is no local radiologist to veto the results. AI developers ultimately need to be held to the same "do no harm" standard as physicians. They should be held accountable, on grounds of negligence, for the unacceptably bad medical outcomes that foreseeably result from the use of their products.

Patient preferences

A poll in 2017 reported that 65% of American adults feel uncomfortable delegating the task of making of a medical diagnosis to a computer with AI[90]. Research is needed to understand when and how patients will, and if they should, trust radiology decisions made by machines.

While radiology should consider the collective wishes of patients with respect to the use of AI tools in their care, these wishes may not conform to the logic that drives AI models. For example, studies about decision-making in autonomous vehicles (AVs) showed that people approve of utilitarian AVs which would sacrifice their passengers for the greater good if faced with a choice of running over pedestrians or sacrificing their occupants, and they would like others to buy them. On the other hand, they themselves preferred to travel in AVs that protect their passengers at all costs[91]. Adding complexity, recent research indicates that norms surrounding AI are culturally variable across the world[92], suggesting that a one-size-fits-all approach will often be impossible.

Similar ambivalence in public attitudes towards radiology AI is likely. Will the public accept imperfections in AI-driven radiology as it relates to individuals, in favor of a potential greater good? Or will an individual deciding for themselves or their loved ones have a much lower tolerance for such imperfections? If, for example, medical imaging is purely protocol-driven and algorithm-interpreted, will there still be room for the practice of common sense, and for

balancing individual and population risks related to radiation exposure against specific patient expectations? If AI-driven radiology is acknowledged to be imperfect and rapidly evolving, will the public accept it because it is less-costly or less-labor intensive than human-provided radiology?

Traceability

Traceability is the ability to link things, and to follow the link. It is a crucial factor to ensure patients' and health care providers' trust in these systems. Traceability helps to detect products that do not function as expected, and to assess quality control and implement corrective actions.

The concept applies to multiple parts of software engineering. In radiology AI, a required diagnosis field in a radiology report, such as presence or absence of disease X, could be linked to an AI model that generates that categorization. Once this link is established, one can trace the relationship to verify the categorization has occurred. Similarly, the categorization can be traced back to the AI model that generated it. Traceability in software testing is the ability to trace tests forward and backward, usually using controlled test cases, or running the AI model in a controlled environment to see if it meets specifications. Traceability matrices document relationships among these requirements.

AI and workforce disruption

One of the greatest fears about AI is that humans will lose their jobs because of it[89]. Radiologists are not immune to this possibility, nor to the fear arising from it. This could lead to behaviors and practices in the future designed to ensure the continuing relevance and roles of human practitioners in health care, regardless of whether or not continued direct human involvement is of ultimate benefit to the public.

Much of the current debate about ethical issues surrounding the use of AI in health care centers around the presumption that one of the key roles of humans in AI implementation is to prevent negative consequences from its utilization. It would be perverse to ignore the possibility that humans may not act disinterestedly, and that radiologists have a vested interest in ensuring they are not made entirely redundant by emerging technology and artificial intelligence. Furthermore, in a potential future where radiologists' position in the hierarchy is threatened or diminished in favor of information scientists or other nontraditional medical players, they may feel driven to protect their relevance. Not only is there an ethical imperative to protect patients and the general public from the dangers of "robot-only radiology," there is also a countervailing need for protection against a radiologist or other physician self-interest if it conflicts with the general good.

We simply don't know how patients will interact with robust radiology AI. Parts of it may be widely embraced, and other parts may generate fear and significant pushback. One described behavior is labeled 'liberal eugenics,' where a subset of the population with special knowledge or access to resources may use them to gain some sort of advantage. For example, they might take advantage of an expensive radiology screening AI tool[93].

Much media attention has been paid in recent years to statements suggesting that radiologists will become redundant in a new age of AI interpretation. This has led to fear among many medical students and young doctors that future careers might not be available to them in radiology, resulting in decreasing applications for places on radiology training programs. As understanding grows about likely AI influences on radiological practice, it seems more probable that we may suffer from the consequences of a future shortage of radiologists arising from this fear. This could paradoxically force accelerated implementation of AI solutions due to a reduced available human workforce, regardless of whether this confers population benefit or not.

Resource inequality

AI requires access to large amounts of data, the technology and skills to manage those data, and computer power to train and manage complex AI systems. Smaller or resource-poor hospitals and academic departments may lack these capabilities. Almost certainly some radiology AI will be proprietary, developed by large academic or private health care entities, insurance companies, or large companies with data science expertise but little historical radiology domain knowledge. This may exacerbate disparities in research capacity and services offered.

While financial incentives must be made available to model developers to foster continued research and development, thought must be given to the well-being of resource-poor communities. Affordable access to models proven to improve individual and population health outcomes may be attainable through government or private funding. In addition, radiologists and other users of models should be cognizant of potential biases towards resource-poor communities due to underrepresentation of certain populations or communities during the training and testing processes. Awareness of these biases can promote recognition of issues as they arise during the implementation and utilization of these models. To these ends, the advisory groups of organizations and institutions in charge of monitoring model performance should be composed of people of diverse backgrounds and expertise to ensure adequate representation. Although there is no universally-agreed upon definition of "fairness," it seems a reasonable position to suggest that health care AI tools should make every effort to offer a sufficient degree of equal opportunity and access for all served by the health care system within which it will be deployed, including minority groups[69]. For example, an algorithm that is

very accurate when given very high quality images and not quite as good when used on lower quality images might still be considered ethical, even if unequal. On the other hand, for example, a TB screening algorithm designed for developed world might work poorly in developing countries, or locations with high HIV rates where the inflammatory response to TB causes different features. Using it in that setting might do more harm than good.

Liability

One offshoot of this issue is whether or not AI should be liable for its actions, and if so, how? This is primarily a legal question, though ethics and morality affect the outcome. For the moment, humans will bear ultimate responsibility and liability[81].

In considering ethics of using AI models in medical practice, one must also consider the liabilities when poor patient outcomes occur. Currently, physicians, including radiologists, are held liable in cases where "standard of care" are not provided. In the new era of AI-assisted care, the "standard of care" is still to be determined. In cases where AI is used as a decision aid, it is likely that radiologists will still be considered liable, though it is probable that litigation will also accuse AI product manufacturers. However, as models incorporate large amounts of data, some of which are not human-perceptible, the question will arise as to whether physicians should still be held wholly responsible for bad outcomes or whether responsibility should be shifted partly or wholly to those who produce, market, and sell models. If, for example, low-dose CT images are manipulated by an algorithm to improve image quality, and this processing alters a subtle but important feature the point of not being visible, the liability should surely reside more with the software developer than with the physician using the tool. Engineers, programmers and the company they work in are potentially liable if the outcome over a large amount of data does not demonstrate a similar ROC and specificity. On the other side, as AI extends into technically sophisticated practice, might radiologists be found guilty for not having used it?

Transparency for AI in radiology should have a means to evaluate whether some culpable defect in the model has contributed to poor patient outcomes. Should the hospital or health care system that implements such models be liable? In addition, what happens when the poor patient outcome is a result of a radiologist using his or her own best judgment against the output of an AI model? Today, a question of a radiologist's liability relates to one of negligence: Did the physician behave reasonably under the circumstances? With an autonomous machine and no human at the controls, will the focus be on whether the computer performed as well as it should have[17, 83]? Furthermore, it is conceivable that a radiologist could be considered liable for a poor outcome if she failed to make use of an available AI tool in the diagnostic process.

The legal issues surrounding AI implementation will be complex, and remain somewhat unpredictable. For example, if AI software is not embedded in any device, but resides in an application, it may be argued that it represents a service, rather than a product, and is therefore not subject to product liability legislation. In the EU, medical devices fall under the Product Liability Directive. The new EU Medical Devices Regulation states that “software in its own right, when specifically intended by the manufacturer to be used for one or more of the medical purposes set out in the definition of a medical device, qualifies as a medical device,” and would therefore fall under product liability legislation[69, 94]. A different issue is whether courts may take the view in the future that failure to use an available AI tool in medical care may constitute negligence[69]. With respect to these complex legal issues, much remains to be decided, by practice and case law.

Conflicts of interest

Conflict of interest (COI) is “a set of circumstances that creates a risk that professional judgment or actions regarding a primary interest will be unduly influenced by a secondary interest.”[95, 96] With nascent, evolving markets like those involving radiology AI, it is expected and quite normal that radiologists involved in patient care would also sometimes hold positions in AI startups or more established commercial entities positioning themselves to compete for position in health care. Similar to when an investigator evaluating a new drug has a financial interest in its success, radiologists or administrators who have COIs related to AI products may be managed through remedies such as public disclosure, institutional oversight, divestment, or other measures.

In some cases, the title or position of a physician, nurse, or administrator in a health care system may effectively render their COI as an institutional COI. Addressing this, the American Association of Medical Colleges states that an individual’s “official’s position may convey an authority that is so pervasive or a responsibility for research programs or administration that is so direct that a conflict between the individual’s financial interests and the institution’s human subjects research should…be considered an institutional conflict of interest.”[97]. With institutional conflicts of interest, institutions may need to be creative with additional independent oversight measures to prevent a loss of public confidence.

Individuals or institutions with conflicts of interest in health care should be vigilant to disclose and manage those conflicts[98, 99]. When dealing with AI in health care, those in positions to facilitate disclosures of patient or subject data to third parties not pursuant to patient care, purchase AI agents, or implement models in clinical workflows should be especially careful to manage their conflicts, which may in some cases require them to recuse themselves from such activities.

As radiology incorporates autonomous and intelligent AI products into widespread, demanding clinical practice, those responsible should be able to answer these and other similar questions about the Ethics of this new Practice paradigm:

- What are the patient and provider risks associated with this AI implementation, and what level of human oversight is necessary to mitigate these risks?
- What education and skills are needed to decide whether to apply AI to our patients, and to safely and effectively use it when appropriate
- How do we ensure that testing data accurately reflects the targeted clinical cohort?
- What system/process should we implement to monitor the impact (outcomes, privacy, and unintended discrimination) of AI on our patients, and providers (automation bias)?
- How do we continuously and actively monitor AI driven autonomous and intelligent tools to verify they are working as expected in clinical care?
- What guardrails should we use to determine when, and more importantly when not, to implement autonomous or intelligent mechanical agents?

Conclusion

AI has the potential to improve radiology, help patients, and deliver cost-effective medical imaging. It amplifies complex ethical and societal questions for radiology. It will conceivably change every part of radiology to some degree. Most of these will be positive, but some may be for the worse. The goal should be to obtain as much value as possible from the ethical use of AI in radiology, yet resist the lure to obtain extra monetary gain from unethical uses of radiology data and AI.

Everyone involved with radiology AI has a duty to understand it deeply, appreciate when and how hazards may manifest and be transparent about them, and to do all they can to mitigate any harm they might cause.

AI has dramatically altered the perception of radiology data — their value, how to use them, and how they may be misused. Because AI allows us to obtain more or previously unknown information from images, radiologists have a duty to understand these new situations with their data. Radiologists and the radiology community have a moral duty to use the data we collect and the potential new insights that AI offers to improve the common good, extract more information about patients and their diseases, and improve the practice of radiology.

For radiology, the value of data and of AI will be more situational than absolute. The radiology community has a duty to strengthen helpful systems and institutions to provide the appropriate circumstances for ethical AI to flourish in clinical care, research, population health, and

business. There will be options to make money from radiology data that are legal, but are still unethical and simply should not be done because they potentially harm patients or society.

Radiology should start now to develop codes of ethics and practice for AI. These codes should promote any use which helps patients and the common good, and block use of radiology data and algorithms for financial gain without those two attributes. Establishing these regulations, standards, and codes of conduct to produce ethical AI means balancing the issues with appropriate moral concern. Ensuring ethical AI requires a desire to gain trust from all parties involved. Regulations, standards, and codes of conduct must be agreed upon and continually updated. We need both radiology-centric AI expertise and technology to verify and validate AI products. Paradoxically, some of this technology may contain AI. Key to these codes of conduct will be a continual emphasis for transparency, protection of patients, and vigorous control of data versions and uses. Continuous post implementation monitoring for unintended consequences and quality escapes with formal root cause and corrective action for these must be enforced.

Radiologists are learning about ethical AI at the same time they invent and implement it. Technological changes in AI, and society's response to them, are evolving at a speed and scope which are hard to grasp, let alone manage. Our understanding of ethical concerns and our appropriate response to them shift constantly. To do best by our patients and our communities, we have a moral obligation to consider the ethics of how we use and appreciate data, how we build and operate decision-making machines, and how we conduct ourselves as professionals.

Definitions

- Artificial intelligence (AI) - The science and engineering of making computers behave in ways that, until recently, were thought to require human intelligence.
- Machine learning (ML) - Algorithms whose performance changes, and ideally improves, as they are exposed to more data. Though AI is the more common term, ML is more accurate for current techniques.
- Supervised ML - A type of ML for which the algorithm changes based on data with known labels. In clinical radiology to evaluate medical images, supervised ML is a repetitive process to match images to existing labels.
- Unsupervised ML - In unsupervised ML, the algorithm is fed an unlabeled dataset (i.e. one without answers). In this case the algorithm groups image findings into clusters based on one or more features it “learns”.
- Deep learning - A type of ML that uses multiple layers of inputs and outputs.
- Neural network - A subset of deep learning that has proved good at making predictions about images
- Algorithm - Computer code that defines the actions that will be performed on input data
- Model - The result of training an algorithm on a dataset. Each time the same algorithm is trained on a different dataset, or a different algorithm is trained with the same dataset, a new model results. Once a model is trained, it runs much faster and requires much less compute power, as long as the input images are similar to the training dataset.
- Bias - A systematic deviation from the truth.
- Variance - A random deviation from the truth.

References

1. Kohli M, Prevedello LM, Filice RW, Geis JR (2017) Implementing Machine Learning in Radiology Practice and Research. Am J Roentgenol 1–7. https://doi.org/10.2214/AJR.16.17224
2. Erickson BJ, Korfiatis P, Akkus Z, Kline TL (2017) Machine Learning for Medical Imaging. RadioGraphics 37:505–515. https://doi.org/10.1148/rg.2017160130
3. García-Pedrajas N, Ortiz-Boyer D, del Castillo Gomariz R, Martínez C (2005) Cascade Ensembles. pp 97–115
4. Floridi L, Taddeo M (2016) What is data ethics? Philos Trans R Soc Math Phys Eng Sci 374:20160360. https://doi.org/10.1098/rsta.2016.0360
5. Mittelstadt BD, Floridi L (2016) The Ethics of Big Data: Current and Foreseeable Issues in Biomedical Contexts. Sci Eng Ethics 22:303–341. https://doi.org/10.1007/s11948-015-9652-2
6. Li Y (Boni), James L, McKibben J (2016) Trust between physicians and patients in the e-health era. Technol Soc 46:28–34. https://doi.org/10.1016/j.techsoc.2016.02.004
7. Obermeyer Z, Emanuel EJ (2016) Predicting the Future — Big Data, Machine Learning, and Clinical Medicine. N Engl J Med 375:1216–1219. https://doi.org/10.1056/NEJMp1606181
8. Char DS, Shah NH, Magnus D (2018) Implementing Machine Learning in Health Care — Addressing Ethical Challenges. N Engl J Med 378:981–983. https://doi.org/10.1056/NEJMp1714229
9. Cabitza F, Rasoini R, Gensini GF (2017) Unintended Consequences of Machine Learning in Medicine. JAMA 318:517–518. https://doi.org/10.1001/jama.2017.7797
10. European Group on Ethics in Science and New Technologies Statement on Artificial Intelligence, Robotics and “Autonomous Systems.” European Commission
11. (2017) Association for Computing Machinery 2018 Code of Ethics and Professional Conduct, Draft 3. In: ACM Ethics. https://ethics.acm.org/2018-code-draft-3/. Accessed 20 Jan 2019
12. IEEE Global Initiative Ethically Aligned Design, Version 2 (EADv2) | IEEE Standards Association. Institute of Electrical and Electronics Engineers
13. The Montreal Declaration for a Responsible Development of Artificial Intelligence: a participatory process. Montreal Declaration for Responsible AI
14. Bill Text - AB-375 Privacy: personal information: businesses. https://leginfo.legislature.ca.gov/faces/billTextClient.xhtml?bill_id=201720180AB375. Accessed 3 Jan 2019
15. Ghosh D (2018) What You Need to Know About California’s New Data Privacy Law. Harv. Bus. Rev.
16. General Data Protection Regulation (GDPR) – Final text neatly arranged. In: Gen. Data Prot. Regul. GDPR. https://gdpr-info.eu/. Accessed 3 Jan 2019
17. European Parliament (2017) Civil Law Rules on Robotics
18. Protection of Human Subjects
19. Kesner A, Laforest R, Otazo R, et al (2018) Medical imaging data in the digital innovation age. Med Phys 45:e40–e52. https://doi.org/10.1002/mp.12794
20. Rubin DL, Kahn CE (2016) Common Data Elements in Radiology. Radiology 161553. https://doi.org/10.1148/radiol.2016161553

21. Shie C-K, Chuang C-H, Chou C-N, et al (2015) Transfer representation learning for medical image analysis. In: 2015 37th Annual International Conference of the IEEE Engineering in Medicine and Biology Society (EMBC). IEEE, Milan, pp 711–714
22. Ravishankar H, Sudhakar P, Venkataramani R, et al (2017) Understanding the Mechanisms of Deep Transfer Learning for Medical Images. ArXiv170406040 Cs
23. Balloch JC, Agrawal V, Essa I, Chernova S (2018) Unbiasing Semantic Segmentation For Robot Perception using Synthetic Data Feature Transfer. ArXiv180903676 Cs
24. Goodfellow I, Pouget-Abadie J, Mirza M, et al (2014) Generative Adversarial Nets. In: Ghahramani Z, Welling M, Cortes C, et al (eds) Advances in Neural Information Processing Systems 27. Curran Associates, Inc., pp 2672–2680
25. Perez L, Wang J (2017) The Effectiveness of Data Augmentation in Image Classification using Deep Learning. ArXiv171204621 Cs
26. Torralba A, Efros AA (2011) Unbiased look at dataset bias. In: CVPR 2011. pp 1521–1528
27. Wendler D (2006) One-time general consent for research on biological samples. BMJ 332:544–547
28. Smith J (2018) European Data Protection Board - Members. In: Eur. Data Prot. Board - Eur. Comm. https://edpb.europa.eu/about-edpb/board/members_en. Accessed 20 Jan 2019
29. Mission – Medical Data Donors. http://www.medicaldatadonors.org/index.php/mission/. Accessed 21 Jan 2019
30. Canada. Supreme Court (1992) McInerney v. MacDonald. Dom Law Rep 93:415–431
31. (2007) Information Governance of the Interoperable EHR | Canada Health Infoway. Canada Health Infoway Inc.
32. (2014) Public Hospitals Act
33. (2010) Tri-council policy statement. Ethical Conduct for Research Involving Humans. Government of Canada
34. Balthazar P, Harri P, Prater A, Safdar NM (2018) Protecting Your Patients' Interests in the Era of Big Data, Artificial Intelligence, and Predictive Analytics. J Am Coll Radiol 15:580–586. https://doi.org/10.1016/j.jacr.2017.11.035
35. (2018) Algorithms in decision-making. House of Commons, United Kingdom Parliament
36. Cloud AutoML | AutoML. In: Google Cloud. https://cloud.google.com/automl/docs/. Accessed 22 May 2019
37. fastMRI Dataset. https://fastmri.med.nyu.edu/. Accessed 22 May 2019
38. CheXpert: A Large Dataset of Chest X-Rays and Competition for Automated Chest X-Ray Interpretation. https://stanfordmlgroup.github.io/competitions/chexpert/. Accessed 22 May 2019
39. Mikk KA, Sleeper HA, Topol EJ (2017) The Pathway to Patient Data Ownership and Better Health. JAMA 318:1433–1434. https://doi.org/10.1001/jama.2017.12145
40. Budin-Ljøsne I, Teare HJA, Kaye J, et al (2017) Dynamic Consent: a potential solution to some of the challenges of modern biomedical research. BMC Med Ethics 18:. https://doi.org/10.1186/s12910-016-0162-9
41. Ballantyne A, Schaefer GO (2018) Consent and the ethical duty to participate in health data research. J Med Ethics 44:392–396. https://doi.org/10.1136/medethics-2017-104550
42. Bonawitz K, Eichner H, Grieskamp W, et al (2019) Towards Federated Learning at Scale: System Design. ArXiv190201046 Cs Stat
43. Dubovitskaya A, Xu Z, Ryu S, et al (2018) Secure and Trustable Electronic Medical Records

Sharing using Blockchain. AMIA Annu Symp Proc 2017:650–659
44. Azaria A, Ekblaw A, Vieira T, Lippman A (2016) MedRec: Using Blockchain for Medical Data Access and Permission Management. In: 2016 2nd International Conference on Open and Big Data (OBD). IEEE, Vienna, Austria, pp 25–30
45. Haug CJ (2017) Whose Data Are They Anyway? Can a Patient Perspective Advance the Data-Sharing Debate? N Engl J Med 376:2203–2205. https://doi.org/10.1056/NEJMp1704485
46. Mello MM, Lieou V, Goodman SN (2018) Clinical Trial Participants' Views of the Risks and Benefits of Data Sharing. N Engl J Med 378:2202–2211. https://doi.org/10.1056/NEJMsa1713258
47. Grapevine World Token. https://grapevineworldtoken.io/. Accessed 21 Jan 2019
48. All-of-Us Program Overview. In: Us. https://www.joinallofus.org/en/program-overview. Accessed 19 Feb 2019
49. Warren SD, Brandeis LD (1890) The Right to Privacy. Harv Law Rev 4:193–220. https://doi.org/10.2307/1321160
50. (2019) Protection of Human Subjects
51. Mazura JC, Juluru K, Chen JJ, et al (2012) Facial Recognition Software Success Rates for the Identification of 3D Surface Reconstructed Facial Images: Implications for Patient Privacy and Security. J Digit Imaging 25:347–351. https://doi.org/10.1007/s10278-011-9429-3
52. Demner-Fushman D, Kohli MD, Rosenman MB, et al (2015) Preparing a collection of radiology examinations for distribution and retrieval. J Am Med Inform Assoc ocv080. https://doi.org/10.1093/jamia/ocv080
53. Na L, Yang C, Lo C-C, et al (2018) Feasibility of Reidentifying Individuals in Large National Physical Activity Data Sets From Which Protected Health Information Has Been Removed With Use of Machine Learning. JAMA Netw Open 1:e186040–e186040. https://doi.org/10.1001/jamanetworkopen.2018.6040
54. Carlini N, Liu C, Kos J, et al (2018) The Secret Sharer: Measuring Unintended Neural Network Memorization & Extracting Secrets. ArXiv180208232 Cs. https://doi.org/arXiv:1802.08232v1
55. Song C, Ristenpart T, Shmatikov V (2017) Machine Learning Models that Remember Too Much. ArXiv170907886 Cs. https://doi.org/arXiv:1709.07886
56. Fairness | Machine Learning Crash Course. In: Google Dev. https://developers.google.com/machine-learning/crash-course/fairness/video-lecture. Accessed 18 Feb 2019
57. (2018) Artificial intelligence and medical imaging 2018: French Radiology Community white paper. Diagn Interv Imaging 99:727–742. https://doi.org/10.1016/j.diii.2018.10.003
58. Khullar D (2019) Opinion | A.I. Could Worsen Health Disparities. N. Y. Times
59. Jordan MI, PhD TGD, Storkey A, et al (2008) Dataset Shift in Machine Learning, First Edition edition. The MIT Press, Cambridge, Mass
60. Calvert JS, Price DA, Chettipally UK, et al (2016) A computational approach to early sepsis detection. Comput Biol Med 74:69–73. https://doi.org/10.1016/j.compbiomed.2016.05.003
61. Mao Q, Jay M, Hoffman JL, et al (2018) Multicentre validation of a sepsis prediction algorithm using only vital sign data in the emergency department, general ward and ICU. BMJ Open 8:e017833. https://doi.org/10.1136/bmjopen-2017-017833

62. Geijer H, Geijer M (2018) Added value of double reading in diagnostic radiology,a systematic review. Insights Imaging 9:287–301. https://doi.org/10.1007/s13244-018-0599-0
63. Boers M, Kirwan JR, Wells G, et al (2014) Developing Core Outcome Measurement Sets for Clinical Trials: OMERACT Filter 2.0. J Clin Epidemiol 67:745–753. https://doi.org/10.1016/j.jclinepi.2013.11.013
64. O'Neil C (2016) Weapons of Math Destruction: How Big Data Increases Inequality and Threatens Democracy, 1 edition. Crown, New York
65. Esteva A, Kuprel B, Novoa RA, et al (2017) Dermatologist-level classification of skin cancer with deep neural networks. Nature 542:115–118. https://doi.org/10.1038/nature21056
66. Verma S, Rubin J (2018) Fairness definitions explained. pp 1–7
67. Gilpin LH, Bau D, Yuan BZ, et al (2018) Explaining Explanations: An Overview of Interpretability of Machine Learning. ArXiv180600069 Cs Stat
68. Explainable Artificial Intelligence. https://www.darpa.mil/program/explainable-artificial-intelligence. Accessed 17 Feb 2019
69. Schönberger D (2019) Artificial intelligence in healthcare: a critical analysis of the legal and ethical implications. Int J Law Inf Technol 27:171–203. https://doi.org/10.1093/ijlit/eaz004
70. Responsible AI Practices. In: Google AI. https://ai.google/education/responsible-ai-practices/. Accessed 17 May 2019
71. Pehrsson E (2018) The Meaning of the GDPR Article 22. Eur Union Law Work Pap 31:37
72. (1985) Convention for the Protection of Individuals with regard to Automatic Processing of Personal Data
73. (2017) Big Data, Artificial Intelligence, Machine Learning, and Data Protection. Information Commissioner's Office
74. ARTICLE29 Newsroom - Guidelines on Automated individual decision-making and Profiling for the purposes of Regulation 2016/679 (wp251rev.01) - European Commission. https://ec.europa.eu/newsroom/article29/item-detail.cfm?item_id=612053. Accessed 3 Jun 2019
75. The Ethics of Unpaid Labor and the OSS Community | Ashe Dryden. https://www.ashedryden.com/blog/the-ethics-of-unpaid-labor-and-the-oss-community. Accessed 3 Jun 2019
76. Werdmuller B (2017) Why open source software isn't as ethical as you think it is. In: Ethical Tech. https://words.werd.io/why-open-source-software-isnt-as-ethical-as-you-think-it-is-2e34d85c3b16. Accessed 3 Jun 2019
77. Mirsky Y, Mahler T, Shelef I, Elovici Y (2019) CT-GAN: Malicious Tampering of 3D Medical Imagery using Deep Learning
78. Chuquicusma MJM, Hussein S, Burt J, Bagci U (2017) How to Fool Radiologists with Generative Adversarial Networks? A Visual Turing Test for Lung Cancer Diagnosis. ArXiv171009762 Cs Q-Bio
79. Finlayson SG, Chung HW, Kohane IS, Beam AL (2018) Adversarial Attacks Against Medical Deep Learning Systems. ArXiv180405296 Cs Stat
80. Kim H, Jung DC, Choi BW (2019) Exploiting the Vulnerability of Deep Learning-Based Artificial Intelligence Models in Medical Imaging: Adversarial Attacks. J Korean Soc Radiol 80:259–273. https://doi.org/10.3348/jksr.2019.80.2.259
81. Jacobson PD Medical Liability and the Culture of Technology. Pew Charitable Trusts

82. Vincent J (2019) The problem with AI ethics. In: The Verge. https://www.theverge.com/2019/4/3/18293410/ai-artificial-intelligence-ethics-boards-charters-problem-big-tech. Accessed 17 May 2019
83. Vladeck DC (2014) Machines without principals: Liability rules and artificial intelligence. Wash Law Rev 89:117–150
84. Parasuraman R, Riley V (1997) Humans and Automation: Use, Misuse, Disuse, Abuse. Hum Factors 39:230–253. https://doi.org/10.1518/001872097778543886
85. Lyell D, Coiera E (2017) Automation bias and verification complexity: a systematic review. J Am Med Inform Assoc 24:423–431. https://doi.org/10.1093/jamia/ocw105
86. Goddard K, Roudsari A, Wyatt JC (2012) Automation bias: a systematic review of frequency, effect mediators, and mitigators. J Am Med Inform Assoc JAMIA 19:121–127. https://doi.org/10.1136/amiajnl-2011-000089
87. Fenton JJ, Taplin SH, Carney PA, et al (2007) Influence of Computer-Aided Detection on Performance of Screening Mammography. N Engl J Med 356:1399–1409. https://doi.org/10.1056/NEJMoa066099
88. Lehman CD, Wellman RD, Buist DSM, et al (2015) Diagnostic Accuracy of Digital Screening Mammography With and Without Computer-Aided Detection. JAMA Intern Med 175:1828–1837. https://doi.org/10.1001/jamainternmed.2015.5231
89. Winfield AF, Jirotka M (2018) Ethical governance is essential to building trust in robotics and AI systems. Philos Trans Math Phys Eng Sci 376:
90. Morning Consult (2017) National Tracking Poll 170401. Morning Consult
91. Bonnefon J-F, Shariff A, Rahwan I (2016) The social dilemma of autonomous vehicles | Science. Science 352:1573–1576. https://doi.org/10.1126/science.aaf2654
92. Awad E, Dsouza S, Kim R, et al (2018) The Moral Machine experiment. Nature 1. https://doi.org/10.1038/s41586-018-0637-6
93. Agar N (2004) Liberal Eugenics: In Defence of Human Enhancement, 1 edition. Wiley-Blackwell, Malden, MA
94. Regulation (EU) 2017/745 of the European Parliament and of the Council of 5 April 2017 on medical devices, amending Directive 2001/83/EC, Regulation (EC) No 178/2002 and Regulation (EC) No 1223/2009 and repealing Council Directives 90/385/EEC and 93/42/EEC
95. Steinbrook R (2009) Controlling Conflict of Interest — Proposals from the Institute of Medicine. N Engl J Med 360:2160–2163. https://doi.org/10.1056/NEJMp0810200
96. Institute of Medicine (US) Committee on Conflict of Interest in Medical Research, Education, and Practice (2009) Conflict of Interest in Medical Research, Education, and Practice. National Academies Press (US), Washington (DC)
97. (2018) Protecting Patients, Preserving Integrity, Advancing Health: Accelerating the Implementation of COI Policies in Human Subjects Research. American Association of Medical Colleges
98. Bero L (2017) Addressing Bias and Conflict of Interest Among Biomedical Researchers. JAMA 317:1723–1724. https://doi.org/10.1001/jama.2017.3854
99. Fineberg HV (2017) Conflict of Interest: Why Does It Matter? JAMA 317:1717–1718. https://doi.org/10.1001/jama.2017.1869
